# Persistent fibroblast growth factor 23 signalling in the parathyroid glands for secondary hyperparathyroidism in mice with chronic kidney disease

**DOI:** 10.1038/srep40534

**Published:** 2017-01-17

**Authors:** Kazuki Kawakami, Ai Takeshita, Kenryo Furushima, Masayasu Miyajima, Ikuji Hatamura, Makoto Kuro-o, Yasuhide Furuta, Kazushige Sakaguchi

**Affiliations:** 1 Department of Molecular Cell Biology and Molecular Medicine, Institute of Advanced Medicine, Wakayama Medical University, 811-1 Kimiidera, Wakayama 641-8509, Japan; 2 Laboratory Animal Center, Wakayama Medical University, 811-1 Kimiidera, Wakayama 641-8509, Japan; 3 Division of Molecular Medicine, Kansai University of Health Sciences, Kumatori 590-0482, Japan; 4 Center for Molecular Medicine, Jichi Medical University, Tochigi 329-0498, Japan; 5 Animal Resource Development Unit and Genetic Engineering Team, RIKEN Center for Life Science Technologies, 2-2-3 Minatojima Minami-machi, Chuou-ku, Kobe 650-0047, Japan

## Abstract

Secondary hyperparathyroidism, in which parathyroid hormone (PTH) is excessively secreted in response to factors such as hyperphosphataemia, hypocalcaemia, and low 1,25-dihydroxyvitamin D (1,25(OH)_2_D) levels, is commonly observed in patients with chronic kidney disease (CKD), and is accompanied by high levels of fibroblast growth factor 23 (FGF23). However, the effect of FGF23 on the parathyroid glands (PG) remains controversial. To bind to FGF receptors, FGF23 requires αKlotho, which is highly expressed in the PG. Here, we examined the effects of *Fgfr1–3, αKlotho*, or *Fgfr1–4* ablation specifically in the PG (conditional knockout, cKO). When mice with early to mid-stage CKD with and without cKO were compared, plasma concentrations of calcium, phosphate, FGF23, and 1,25(OH)_2_D did not change significantly. In contrast, plasma PTH levels, which were elevated in CKD mice, were significantly decreased in cKO mice. PG from CKD mice showed augmentation of cell proliferation, which was significantly suppressed by cKO. Parathyroid tissue cultured for 4 days showed upregulation of PTH secretion and cell proliferation in response to FGF23. Both these effects were inhibited by cKO. These findings suggest that FGF23 is a long-term inducer of parathyroid cell proliferation and PTH secretion, and is one cause of secondary hyperparathyroidism in CKD.

The primary regulation of parathyroid hormone (PTH) secretion is mediated by calcium ions that directly bind to calcium-sensing receptors expressed on parathyroid cells^1^. High serum phosphate levels appear to bind to serum calcium and decrease the levels of ionised calcium, resulting in the stimulation of hormone secretion^2^. Serum phosphate also directly affects PTH synthesis by promoting the stability of PTH mRNA^3^. 1,25-dihydroxyvitamin D (1,25(OH)_2_D), the active form of vitamin D synthesised from 25-dihydroxyvitamin D (25(OH)D) in the kidney, is a hormone that regulates parathyroid cell proliferation and PTH synthesis not only directly, but also indirectly by increasing the absorption of calcium and phosphate from the intestine^4^. Calcium and 1,25(OH)_2_D act in co-ordination to suppress PTH gene expression and inhibit parathyroid cell proliferation^5^.

Fibroblast growth factor 23 (FGF23) belongs to a subfamily of endocrine FGF ligands and is secreted from osteoblasts and osteocytes, presumably to regulate renal phosphate reabsorption and vitamin D activation^6^. FGF23 requires αKlotho as a co-receptor for binding to fibroblast growth factor receptors (FGFRs). αKlotho is predominantly expressed in the kidney, the epithelium of the choroid plexus, and the parathyroid glands, and thereby limits the organs targeted by FGF23. The renal function of FGF23 is well studied in mice, and αKlotho ablation results in hyperphosphataemia, aberrant vitamin D metabolism, impaired growth, and complex ageing-like phenotypes^7^. Parathyroid cells express both αKlotho and FGFRs and play an important role in mineral metabolism. However, the effect of FGF23 on the parathyroid glands has been controversial^7^. Acute administration of recombinant FGF23 increases ERK phosphorylation and *Egr1* mRNA expression in murine parathyroid glands^8^, but the physiological function of FGF23 in the parathyroid glands is not fully clarified^7^,^9^,^10^. Some studies have indicated that FGF23 suppresses PTH mRNA expression and hormone secretion *in vitro*^11^, and that FGF23 negatively regulates PTH secretion *in vivo*^12^. However, other studies show contradictory results. *Fgf23* null mice overexpressing human recombinant FGF23 show elevated serum PTH levels despite normal calcium, 1,25(OH)_2_D, and hypophosphataemia^13^, supporting the hypothesis that FGF23 acts on the parathyroid glands to induce PTH production. FGF23 and PTH show a strong positive correlation in patients with X-linked hypophosphataemia who are normocalcaemic and display low to normal levels of 1,25(OH)_2_D^14^. In addition, transgenic overexpression of FGF23 in mice causes secondary hyperparathyroidism^15^,^16^, although this might be partially explained by the suppression of the circulating levels of 1,25(OH)_2_D by FGF23.

Patients with advanced chronic kidney disease (CKD) typically exhibit secondary hyperparathyroidism associated with high serum FGF23, high serum phosphate, and low 1,25(OH)_2_D levels^19^. FGF23 normally suppresses αKlotho expression, while 1,25(OH)_2_D promotes it. The CKD-induced low level of αKlotho expression in the parathyroid glands leads to reduced functionality of the αKlotho/FGFR complex for FGF23 signalling^20^,^21^. Thus, reduced FGF23 signalling may explain the resistance to the putative inhibitory effects of FGF23 on the parathyroid glands, but cannot disprove the involvement of FGF23 in the pathogenesis of secondary hyperparathyroidism. FGF23 is known to be elevated in patients with CKD from the early phase of the disease^19^.

An interesting case was reported in which the *αKlotho* gene was translocated and overexpressed^17^. In this case, the patient had hypophosphataemic rickets, hyperparathyroidism due to parathyroid hyperplasia, and low to normal 1,25(OH)_2_D levels in the setting of hypophosphataemia. PTH levels were high in the absence of low serum calcium. Interestingly, the circulating concentration of αKlotho was markedly increased. To clarify the function of this circulating αKlotho, another research group has generated mice carrying an adeno-associated virus vector that produces an endo-proteolytic cleavage product of αKlotho (cKL)^18^. These mice exhibited overexpression of bone *Fgf23* mRNA and other features resembling those observed in the case with a translocated *αKlotho* gene. These findings suggest that cKL increases the production of bone-derived FGF23, and that elevated FGF23 and cKL levels cause the observed phenotypes. The hyperparathyroidism in this patient and mice is speculated to be caused by enhanced FGF23 signalling through the αKlotho/FGFR complex^18^.

To explain these experimental and clinical findings related to the function of FGF23 and αKlotho in the parathyroid glands, we hypothesised that FGF23 transduces a signal to induce the proliferation of parathyroid cells via the αKlotho/FGFR complex. To test this hypothesis, we used a long-term tissue culture system and a strategy of conditionally knocking out the *αKlotho* or *Fgfr* genes specifically from murine parathyroid cells to minimise the extra-parathyroidal effect of FGF23 signalling ablation on mineral metabolism.

## Results

### Expression of FGFRs and αKlotho in the parathyroid glands

**Figure 1:**
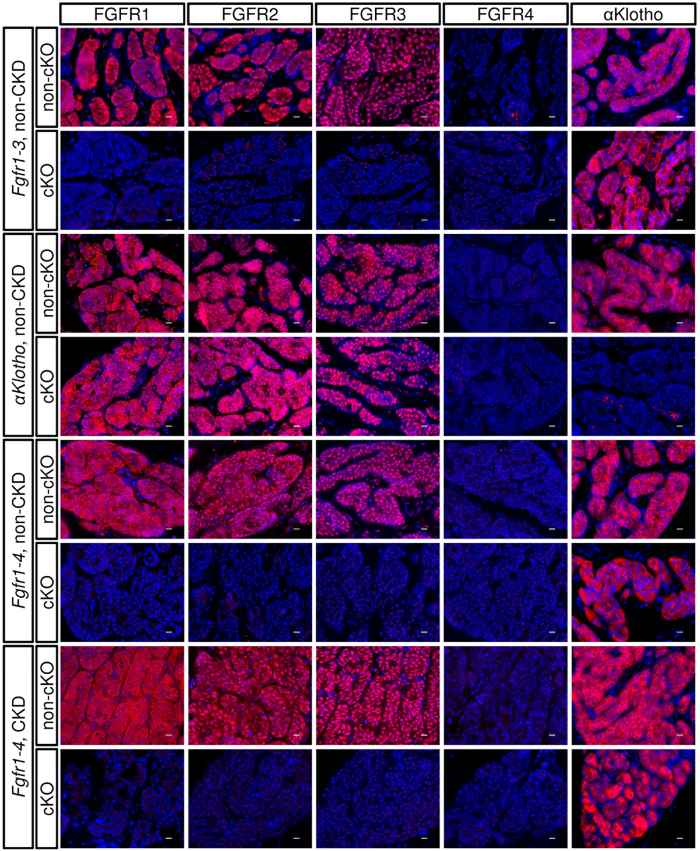
Expression of FGFRs (FGFR1, FGFR2, FGFR3, and FGFR4) and αKlotho in normal and genetically engineered parathyroid glands. A specific gene or genes were conditionally manipulated in the parathyroid glands by mating *Fgfr1–3*^*flox/flox*^, *αKlotho*^*flox/flox*^, or *Fgfr1–4*^*flox/flox*^ mice with *PTH-Cre* mice (cKO) or without mating (non-cKO). The mice were treated with heminephrectomy plus a high-phosphate diet (CKD) or not treated (non-CKD) using the protocol described in the Methods section. Paraffin-embedded thyro-parathyroid glands were sectioned and immunostained (red) using an indirect immunofluorescence technique as described in the Methods section. Nuclei were stained with DAPI (blue). Scale bars: 10 μm.

To examine the extent of the expression of four *Fgfr* genes, we performed histochemical analyses of each of their proteins. We found that parathyroid cells abundantly express FGFR1, FGFR2, and FGFR3, but not FGFR4 (Fig. 1). αKlotho was clearly expressed.

We examined the specificity of the anti-FGFR4 antibody. The antibody specifically detected FGFR4 expression in the liver of wild-type mice, but not *Fgfr4* knockout (KO) mice (Supplementary Fig. S6). However, since the antibody for FGFR4 might not be adequately sensitive for immuno-detection in the parathyroid glands, we decided to generate not only an *Fgfr1–3* conditional KO (cKO) mouse (*Fgfr1–3*^*flox/flox*^*;PTH-Cre*) but also *an Fgfr1–4* cKO mouse (*Fgfr1–4*^*flox/flox*^*;PTH-Cre*) to examine the effect of total FGFR ablation from parathyroid cells. In addition, to examine whether FGF23 is involved in parathyroid cell proliferation, we generated mice with parathyroid-specific ablation of *αKlotho (αKlotho*^*flox/flox*^*;PTH-Cre*). All cKO mice were born alive, and as shown below, biochemical markers did not change in any mice with or without cKO under a regular diet.

We also examined FGFR1–4 and αKlotho protein expression in CKD mice (Fig. 1), which were generated as described in the Methods section. When the non-CKD and CKD groups were compared in the *Fgfr1–4*^*flox/flox*^(non-cKO) genotype group, no marked difference was observed in the expression of any FGFR or αKlotho using the same immunohistochemistry conditions, although immunohistochemistry is not strictly a quantitative method.

### Generation of CKD model mice with secondary hyperparathyroidism (CKD mice)

**Figure 2:**
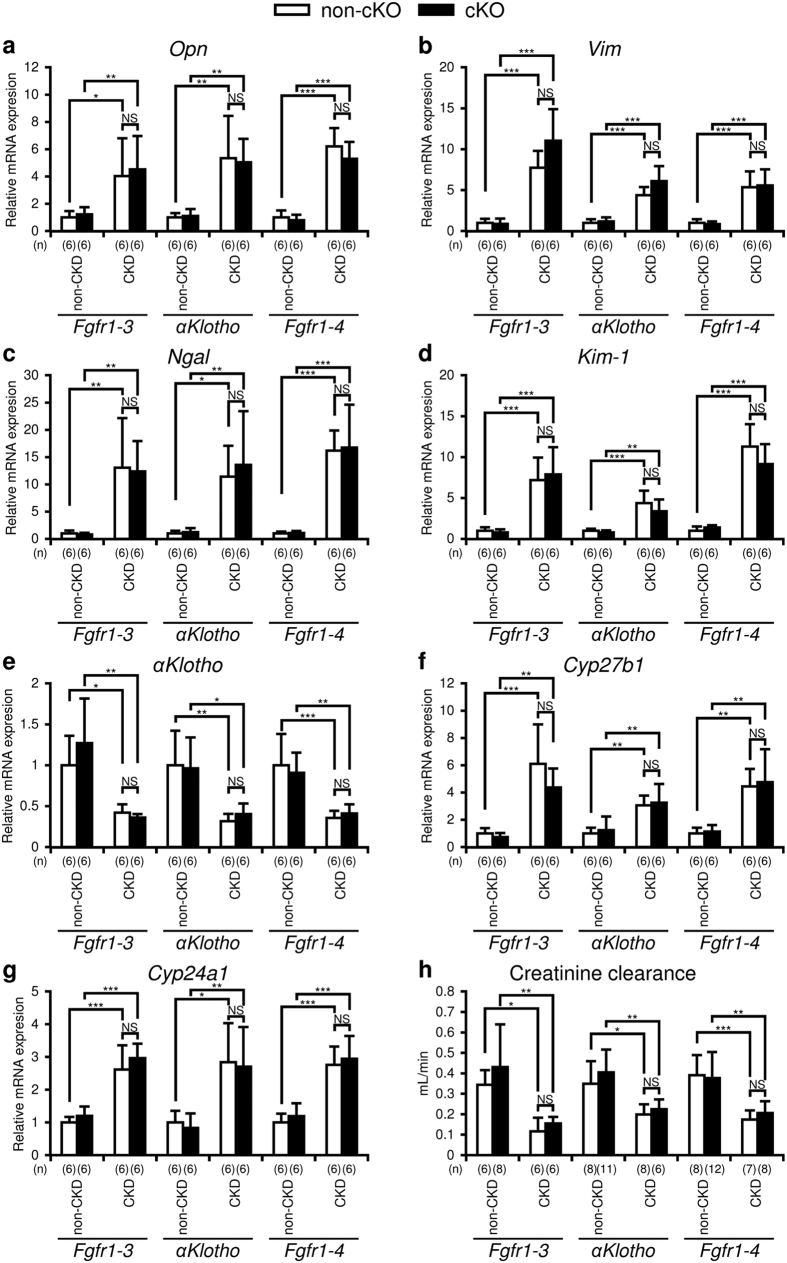
Markers for chronic kidney disease (CKD). Quantitative RT-PCR using kidney RNA to analyse the mRNA levels of osteopontin (*Opn*) (**a**), vimentin (*Vim*) (**b**), neutrophil gelatinase-associated lipocalin (*Ngal)* (**c**), kidney injury molecule 1 (*Kim-1)* (**d**), *αKlotho* (**e**), *Cyp27b1* (**f**), and *Cyp24a1* (**g**) was carried out as described in the Methods section in mice with or without treatment (heminephrectomy plus high-phosphate diet) for CKD, and with (black bar) or without (white bar) parathyroid-specific genetic cKO of *Fgfr1–3, αKlotho*, or *Fgfr1–4*. Creatinine clearance (**h**) was also analysed as described in the Methods section. Values are expressed as mean ± s.d. n = 6 per group for panels a–g, and n = 6–12 for panel h; *P < 0.05, **P < 0.01, ***P < 0.001. NS, not significantly different (P > 0.05).

CKD model mice were generated by heminephrectomy at 8 weeks of age followed by a high-phosphate and low-calcium diet (2.0% inorganic phosphate and 0.4% calcium) for 12 weeks starting at 12 weeks of age, as described in the Methods section^22^. The diet between 4 and 12 weeks of age contained 0.83% inorganic phosphate and 1.07% calcium (regular diet). The non-CKD mice were fed the regular diet without heminephrectomy. At 24 weeks of age, all mice were sacrificed to collect blood and organ specimens. To evaluate the extent of kidney damage of the mice with three different genetic ablations (cKO of *Fgfr1–3, αKlotho*, and *Fgfr1–4*), we carried out quantitative RT-PCR (qRT-PCR) of osteopontin (*Opn*), vimentin (*Vim*), neutrophil gelatinase-associated lipocalin (*Ngal*), kidney injury molecule 1 (*Kim-1*), and *αKlotho* using RNA extracted from the kidney (Fig. 2a–e). *Opn*, a marker for vascular calcification^23^, *Vim*, a marker for kidney fibrosis^24^, and *Ngal* and *Kim-1*, markers for tubular damage^25^, increased significantly in the CKD model mice compared with the control mice. *αKlotho*, another marker for kidney damage^26^, also decreased significantly in the CKD mice. We also examined the mRNA expression of *Cyp27b1* and *Cyp24a1*, vitamin D-metabolising enzymes, in the kidney. They were both enhanced in the CKD groups of all three different genetic ablations (Fig. 2f and g). The expression of these markers did not change in either non-CKD or CKD mice following parathyroid-specific gene ablation of *Fgfr1–3, αKlotho*, or *Fgfr1–4*.

We calculated creatinine clearance by measuring urine volume and the concentrations of plasma and urinary creatinine. Creatine clearance decreased by 45–65% in CKD mice compared with the controls (Fig. 2h) but was not significantly different between the cKO and non-cKO mice in either the non-CKD or CKD group.

### Effects of *Fgfr* cKO and *αKlotho* cKO on biochemical parameters

**Figure 3:**
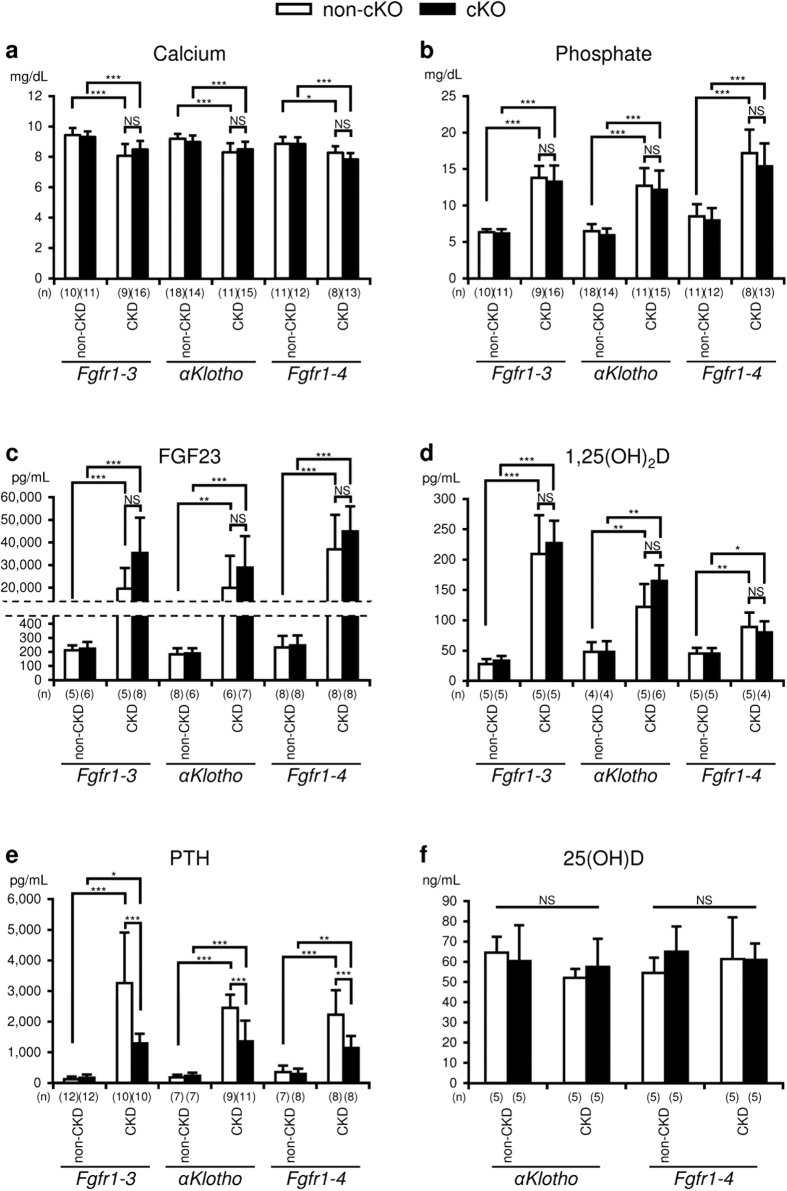
Biochemical analysis of plasma. Plasma levels of calcium (**a**), phosphate (**b**), FGF23 (**c**), 1,25 dihydroxyvitamin D (1,25(OH)_2_D) (**d**), parathyroid hormone (PTH) (**e**), and 25 hydroxyvitamin D (25(OH)D) (**f**) were measured as described in the Methods section in mice with or without CKD and with (black bar) or without (white bar) parathyroid-specific cKO of *Fgfr1–3, αKlotho*, or *Fgfr1–4*. Values are expressed as mean ± s.d. n = 7–18 per group for plasma Ca, Pi, and PTH, and n = 4–8 per group for plasma FGF23 and 1,25D; *P < 0.05, **P < 0.01, ***P < 0.001. NS, not significantly different (P > 0.05).

We measured the concentrations of plasma calcium, phosphate, FGF23, 1,25(OH)_2_D, PTH, and 25(OH)D (Fig. 3). In the non-cKO groups, plasma calcium levels significantly decreased in CKD mice compared with non-CKD mice (control): *Fgfr1–3*^*flox/flox*^, control (9.4 ± 0.5 mg/dL, n = 10) vs CKD (8.1 ± 0.8 mg/dL, n = 9), P < 0.001; *αKlotho*^*flox/flox*^, control (9.2 ± 0.3 mg/dL, n = 18) vs CKD (8.3 ± 0.6 mg/dL, n = 11), P < 0.001; *Fgfr1–4*^*flox/flox*^, control (8.9 ± 0.5 mg/dL, n = 11) vs CKD (8.3 ± 0.4 mg/dL, n = 8), P < 0.05. Plasma phosphate levels were significantly increased in CKD mice: *Fgfr1–3*^*flox/flox*^, control (6.3 ± 0.4 mg/dL, n = 10) vs CKD (13.8 ± 1.6 mg/dL, n = 9), P < 0.001; *αKlotho*^*flox/flox*^, control (6.5 ± 1.0 mg/dL, n = 18) vs CKD (12.7 ± 2.4 mg/dL, n = 11), P < 0.001; *Fgfr1–4*^*flox/flox*^, control (8.5 ± 1.7 mg/dL, n = 11) vs CKD (17.2 ± 3.2 mg/dL, n = 8), P < 0.001. Plasma FGF23 levels were significantly elevated in CKD mice: *Fgfr1–3*^*flox/flox*^, control (212.0 ± 34.2 pg/mL, n = 5) vs CKD (19531.1 ± 9232.3 pg/mL, n = 5), P < 0.001; *αKlotho*^*flox/flox*^, control (182.0 ± 43.6 pg/mL, n = 8) vs CKD (19949.4 ± 14188.7 pg/mL, n = 6), P < 0.01; *Fgfr1–4*^*flox/flox*^, control (232.0 ± 82.7 pg/mL, n = 8) vs CKD (37005.2 ± 15197.4 pg/mL, n = 8), P < 0.001, corresponding to the levels of plasma phosphate. 1,25(OH)_2_D levels were also increased in CKD mice: *Fgfr1–3*^*flox/flox*^, control (27.7 ± 8.5 pg/mL, n = 5) vs CKD (209.3 ± 63.8 pg/mL, n = 5), P < 0.001; *αKlotho*^*flox/flox*^, control (48.0 ± 15.8 pg/mL, n = 4) vs CKD (122.2 ± 37.7 pg/mL, n = 5), P < 0.01; *Fgfr1–4*^*flox/flox*^, control (45.0 ± 9.5 pg/mL, n = 5) vs CKD (88.9 ± 24.0 pg/mL, n = 4), P < 0.01. Plasma PTH levels increased significantly in CKD mice: *Fgfr1–3*^*flox/flox*^, control (122.6 ± 84.3 pg/mL, n = 12) vs CKD (3264.7 ± 1654.5 pg/mL, n = 10), P < 0.001; *αKlotho*^*flox/flox*^, control (182.6 ± 87.0 pg/mL, n = 7) vs CKD (2452.4 ± 425.9 pg/mL, n = 9) P < 0.001; *Fgfr1–4*^*flox/flox*^, control (357.2 ± 210.8 pg/mL, n = 7) vs CKD (2226.8 ± 801.8 pg/mL, n = 8) P < 0.001. The genetic deletion of *Fgfr1–3, αKlotho*, or *Fgfr1–4* from the parathyroid glands by mating with *PTH-Cre* mice did not affect the plasma levels of calcium, phosphate, FGF23, and 1,25(OH)_2_D in the non-CKD and CKD groups. In contrast to these mineral metabolism parameters, the same genetic deletions significantly suppressed plasma PTH concentrations in the CKD groups: *Fgfr1–3*^*flox/flox*^ (3264.7 ± 1654.5 pg/mL, n = 10) vs *Fgfr1–3*^*flox/flox*^*;PTH-Cre* (1290.9 ± 312.2 pg/mL, n = 10), P < 0.001; *αKlotho*^*flox/flox*^ (2452.4 ± 425.9 pg/mL, n = 9) vs *αKlotho*^*flox/flox*^*;PTH-Cre* (1357.3 ± 675.4 pg/mL, n = 11), P < 0.001; *Fgfr1–4*^*flox/flox*^ (2226.8 ± 801.8 pg/mL, n = 8) vs *Fgfr1–4*^*flox/flox*^*;PTH-Cre* (1144.3 ± 388.3 pg/mL, n = 8), P < 0.001, even though the suppressed levels were higher than those in the non-CKD groups. These higher than normal levels of PTH in cKO mice suggest the involvement of multiple factors, other than FGF23/αKlotho/FGFR signalling, in the pathogenesis of secondary hyperparathyroidism. The basal concentration of PTH in the non-CKD groups was not affected by these genetic deletions.

Since the levels of 1,25(OH)_2_D were high in CKD mice, we also measured 25(OH)D levels in the *αKlotho* and *Fgfr1–4* groups: *αKlotho*^*flox/flox*^, control (64.6 ± 7.8 ng/mL, n = 5) vs *αKlotho*^*flox/flox*^, CKD (52.0 ± 4.4 ng/mL, n = 5), *αKlotho*^*flox/flox*^*;PTH-Cre*, control (60.4 ± 17.7 ng/mL, n = 5), *αKlotho*^*flox/flox*^*;PTH-Cre*, CKD (57.4 ± 13.9 ng/mL, n = 5); *Fgfr1–4*^*flox/flox*^, control (54.4 ± 7.6 ng/mL, n = 5) vs *Fgfr1–4*^*flox/flox*^, CKD (61.4 ± 20.6 ng/mL, n = 5), *Fgfr1–4*^*flox/flox*^*;PTH-Cre*, control (65.0 ± 12.5 ng/mL, n = 5), *Fgfr1–4*^*flox/flox*^*;PTH-Cre*, CKD (61.0 ± 8.1 ng/mL, n = 5). These values were not significantly different in comparisons between any of the two groups in either the *αKlotho* or *Fgfr1–4* group.

### Histochemical analyses of parathyroid tissue taken from mice with various genetic and kidney conditions

**Figure 4:**
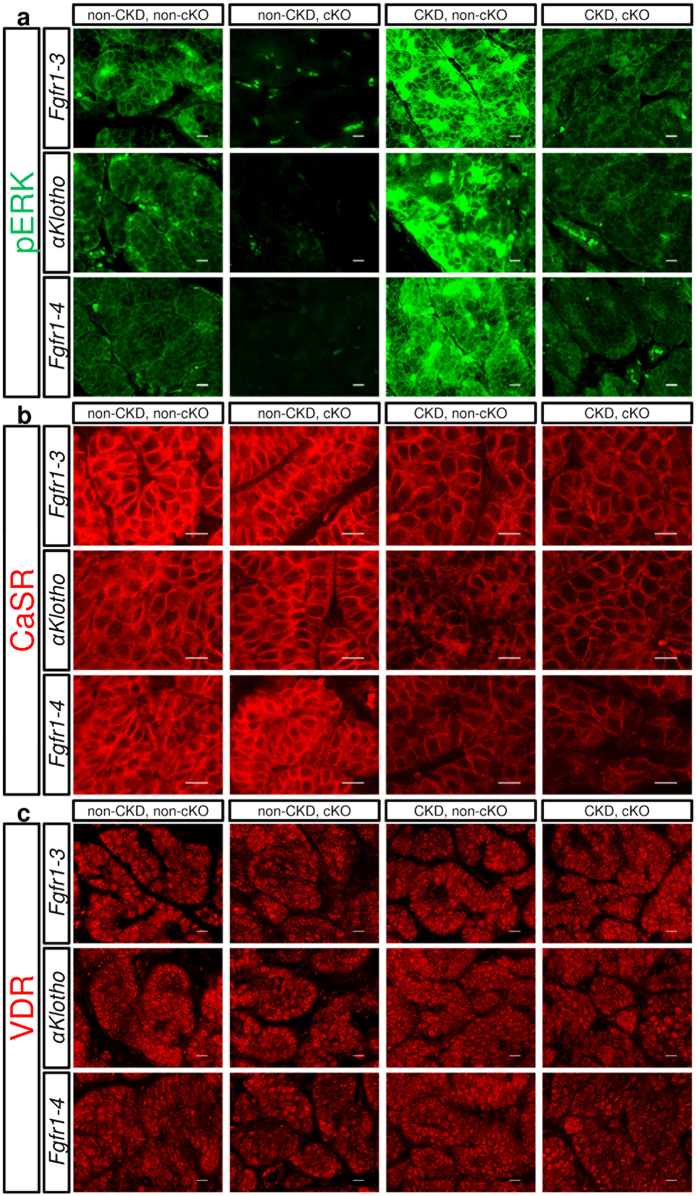
Histochemical analysis of phosphorylated ERK1/2 (pERK) (**a**; green), calcium sensing receptor (CaSR) (**b**; red), and vitamin D receptor (VDR) (**c**; red). Paraformaldehyde-treated paraffin-embedded thyro-parathyroid glands were sectioned and stained for pERK, CaSR, and VDR using the indirect immunofluorescence staining technique as described in the Methods section. *Fgfr1–3*^*flox/flox*^, *αKlotho*^*flox/flox*^, or *Fgfr1–4*^*flox/flox*^ mice were mated (cKO) or not mated (non-cKO) with *PTH-Cre* mice, and treated (heminephrectomy plus high-phosphate diet) or not treated for CKD, as described in the Methods section. Scale bars: 10 μm.

To examine whether parathyroid cells were stimulated to proliferate *in vivo*, we first semi-quantitatively examined the levels of ERK phosphorylation in parathyroid tissue sections using an immunohistochemical method under the same conditions (Fig. 4a). ERK phosphorylation was slightly elevated in the non-CKD and non-cKO groups as compared with the non-CKD and cKO groups, whereas ERK was strongly phosphorylated in the CKD and non-cKO groups. This strong phosphorylation of ERK was suppressed to the level of the non-CKD and non-cKO groups by cKO of *Fgfr1–3, αKlotho*, or *Fgfr1–4*.

Calcium-sensing receptor (CaSR) and vitamin D receptor (VDR) are also involved in the regulation of parathyroid cell proliferation; therefore, we examined their expression levels in parathyroid tissue sections. CaSR expression (Fig. 4b) appeared slightly suppressed in the CKD groups, but was not affected by genetic deletion of *Fgfr1–3, Fgfr1–4*, or *αKlotho*. VDR expression (Fig. 4c) was not affected by the status of kidney function or the genetic deletions in our model. These semi-quantitative analyses might not be sufficient to compare the expression levels in different groups precisely, but the small size of the parathyroid glands did not allow us to carry out quantitative analysis such as western blotting.

**Figure 5:**
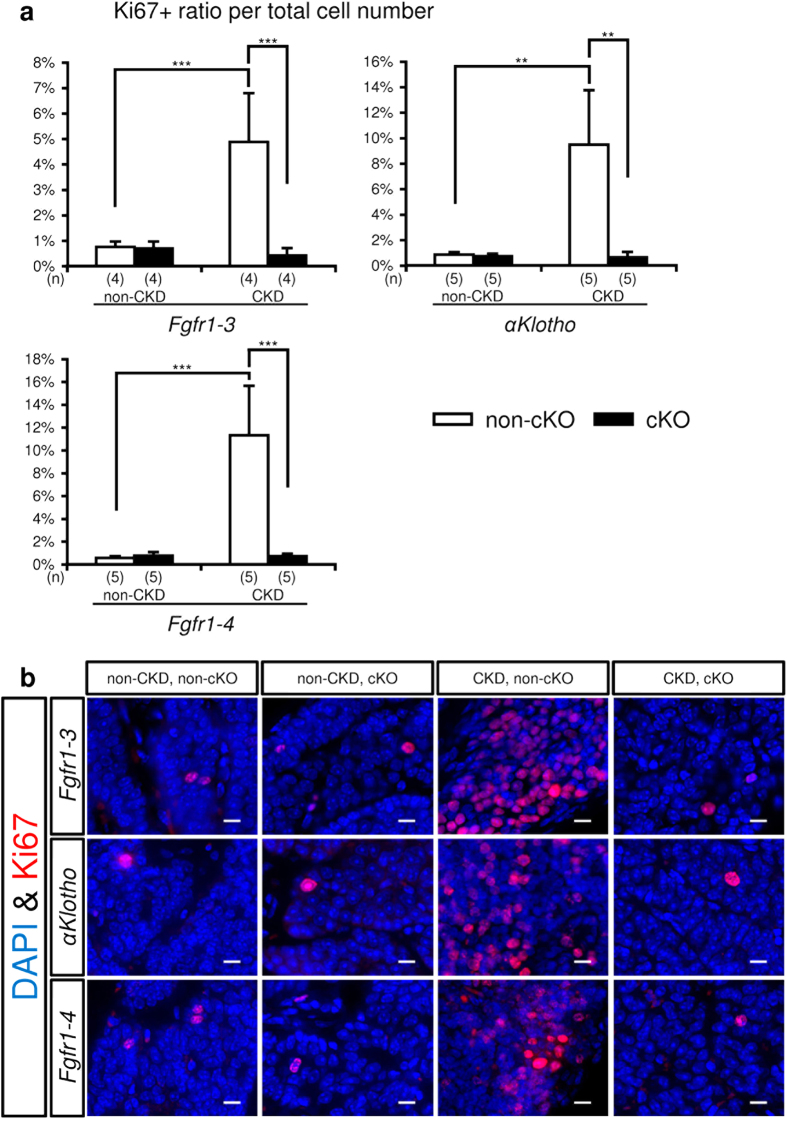
Mitogenic analysis of parathyroid cells. Parathyroid glands were derived from *Fgfr1–3*^*flox/flox*^, *αKlotho*^*flox/flox*^, and *Fgfr1–4*^*flox/flox*^ mice that were mated with *PTH-Cre* mice (cKO, black bar) or not mated (non-cKO, white bar), and treated or not treated for CKD. Thyro-parathyroid glands were dissected from both sides under a microscope, and treated with paraformaldehyde for preparing paraffin-embedded blocks. Each thyro-parathyroid gland was sectioned serially at 6 μm throughout the tissue, and one in six sections was stained for Ki67, a cell proliferation marker, with DAPI counterstaining for nuclei. The total number of Ki67+ and DAPI+ cells was counted only in parathyroid tissue, and their ratio was calculated. The average ratio of the Ki67+/DAPI+ cell numbers in sections of parathyroid glands from both sides was taken as representing one mouse. (**a**) Summary of the ratio of Ki67+ parathyroid cells. Values are expressed as mean ± s.d. n = 4–5 per group. **P < 0.01, ***P < 0.001. (**b**) Representative micrographs of Ki67+ parathyroid cells (red) with DAPI staining (blue) for nuclei. Scale bars: 10 μm.

Ki67 is one of the most reliable markers of cell cycle progression. We counted the number of cells expressing this marker in tissue sections of parathyroid glands taken from mice with various genetic and kidney conditions (Fig. 5). As described in the Methods section, the number of Ki67+ cells and total parathyroid cell number were counted evenly in whole parathyroid glands. The Ki67+ cell ratio per total cell number increased in mice with CKD alone, and this ratio was completely suppressed to the basal level by genetic deletion of *Fgfr1–3, Fgfr1–4*, or *αKlotho*.

### Effect of FGF23 on PTH secretion

To examine whether FGF23 influences the long-term secretion of PTH, we set up a long-term tissue culture system that enabled parathyroid cells to survive and continuously secrete PTH for up to 5 days, as described in the Methods section. Using this system, we could observe long-term PTH secretion regulated by a physiological range of calcium concentrations (0.95 vs 1.5 mM) (Supplementary Fig. S7). We also examined parathyroid cell viability by histochemically checking the percentage of apoptotic parathyroid cells at the end of the 4-day incubation period (Supplementary Fig. S8). The percentage of apoptotic cells was less than 0.5% under all the conditions examined. The ratio did not significantly change in the presence or absence of *Fgfr1–3* or *αKlotho* cKO, or after incubation with FGF23.

**Figure 6:**
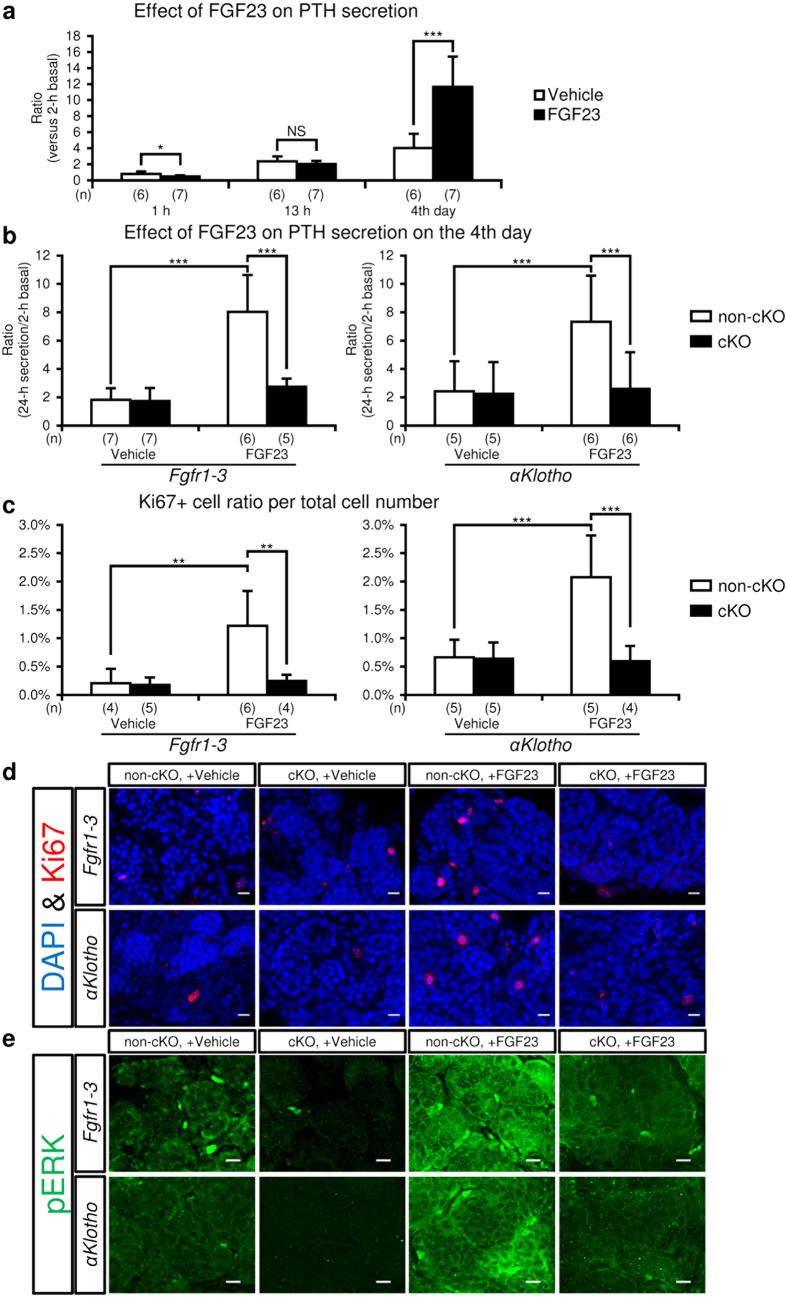
Effect of FGF23 on PTH secretion (**a** and **b**) and expression of cell proliferation markers (**c,d** and **e**) in cultured parathyroid glands. A parathyroid gland with some attached thyroid tissue was micro-dissected from mice under a stereomicroscope, coated with collagen gel, and incubated in MEM with FGF23 (100 ng/mL) or vehicle (saline) for 4 days as described in the Methods section. (**a**) Time-course of the effect of FGF23 on PTH secretion from normal parathyroid glands. The amount of PTH secreted after 1- and 13-h incubation with (black bar) or without (white bar) FGF23 (100 ng/mL) is expressed as a cumulative value, while the value on day 4 represents the amount of PTH secreted in 24 h. PTH secreted in the culture medium versus baseline medium PTH secretion in the initial 2-h incubation period was regarded as representing PTH secretion under the specific condition. (**b**) Effect of FGF23 on PTH secretion on day 4 in the parathyroid glands from mice with or without cKO of *Fgfr1–3* or *αKlotho. Fgfr1–3*^*flox/flox*^ and *αKlotho*^*flox/flox*^ mice were mated with *PTH-Cre* mice (cKO, black bar) or not mated (non-cKO, white bar). PTH secretion experiments were carried out as described above. (**c**) Percentage of Ki67+ cell number in cultured parathyroid glands. The Ki67+ and DAPI+ cell numbers were detected and counted as described in the Methods section. (**d**) Representative micrographs of Ki67 expression (red) with DAPI staining (blue) for nuclei in tissue sections of cultured parathyroid glands. (**e**) Representative micrographs of staining for phosphorylated ERK1/2 (pERK; green) in parathyroid tissue sections. Scale bars: 10 μm. Values are expressed as mean ± s.d. *P < 0.05, **P < 0.01, ***P < 0.001. NS, not significantly different (P > 0.05).

To study the time course of the effect of FGF23 on PTH secretion, parathyroid glands were incubated with medium containing 100 ng/mL FGF23 (Fig. 6a) for 4 days after measurement of basal PTH secretion. The medium was collected and replaced with fresh medium at 1 h, 13 h, and 24 h, and every 24 h thereafter. The concentrations of PTH in the media at 1 h and 13 h after initiation of incubation were measured separately, enabling us to calculate the amount of PTH secreted during the initial 1 h, 12 h following the 1-h period, and the whole 13-h incubation period. PTH secretion on day 4 indicates the amount of PTH secreted in 24 h on day 4. The ratio of PTH secretion at each time point versus 2-h basal secretion is shown in Fig. 6a: 1 h (control, 0.78 ± 0.31, n = 6 vs FGF23, 0.48 ± 0.13, n = 7, P < 0.05); 13 h (control, 2.37 ± 0.68, n = 6 vs FGF23, 2.01 ± 0.44, n = 7, P = 0.28); day 4 (control, 4.02 ± 1.77, n = 6 vs FGF23, 11.67 ± 3.76, n = 7, P < 0.001). The values for the 12-h period between the 1 h and 13 h time points were as follows: control, 1.59 ± 0.61, n = 6 vs FGF23, 1.53 ± 0.40, n = 7, P = 0.84). PTH secretion was significantly reduced by FGF23 after 1-h incubation, unchanged after 13-h incubation, and markedly increased on day 4. These results also show that the PTH-reducing effect of FGF23 was almost complete during the first 1 h of incubation.

### Effects of *Fgfr* cKO and *αKlotho* cKO on cultured parathyroid tissue

When parathyroid glands were incubated with 100 ng/mL FGF23, 24-h PTH secretion on day 4 increased by approximately four-fold compared with basal secretion in the absence of FGF23. This elevated secretion was almost completely suppressed to the basal level by cKO of *Fgfr1–3* or *αKlotho* (Fig. 6b). The cultured parathyroid glands were also sectioned and stained for Ki67. The ratio of Ki67+ versus DAPI+ cell numbers per parathyroid gland of each mouse was analysed as described in the Methods section. The ratio of Ki67+ cells increased after incubation with FGF23 by approximately three-fold, and this increase was suppressed to the basal level in mice with cKO of *Fgfr1–3* or *αKlotho* (Fig. 6c and d). These sections were also stained for phosphorylated ERK (Fig. 6e). Corresponding to the results for Ki67, ERK was strongly phosphorylated when the glands were incubated with FGF23, and ERK phosphorylation was almost completely suppressed to the basal level in mice with cKO of *Fgfr1–3* or *αKlotho.*

## Discussion

In CKD, FGF23 production and release from osteoblasts/osteocytes are elevated before the increase of PTH or phosphate^27^,^28^. The sensor that triggers bone cells to release FGF23 has not yet been identified. FGF23 compensates for the decrease in the number of nephrons by accelerating the rate of phosphaturia per nephron by inhibiting the reabsorption of phosphate from the proximal tubule, and thus reduces the phosphate load^19^,^29^. The other outstanding features of advanced CKD are low 1,25(OH)_2_D levels, secondary hyperparathyroidism, and hypocalcaemia. FGF23 suppresses the production of 1,25(OH)_2_D from the kidney due to decreased 1α hydroxylation of 25-hydroxy vitamin D. The resulting low concentrations of 1,25(OH)_2_D lessen the suppression of the parathyroid glands by this active form of vitamin D^5^, leading to the enhanced secretion of PTH, and also the induction of hypocalcaemia by decreasing calcium absorption from the colon^19^. The high levels of circulating phosphate in late-stage CKD decrease the concentration of ionised calcium. The eventual reduction of calcium concentration also enhances the secretion of PTH^2^. These main mechanisms of parathyroid regulation in CKD by FGF23, vitamin D, calcium, and phosphate have been confirmed in many studies to date. Thus, secondary hyperparathyroidism has been postulated to be the direct result of low 1,25(OH)_2_D levels and hypocalcaemia^5^,^19^.

However, the direct function of FGF23 in the parathyroid glands has yet to be clarified. There are reports from different groups suggesting either suppressive or stimulatory effects of FGF23 in PTH secretion^11^,^12^,^13^,^15^,^16^,^30^. In general, the factors influencing mineral metabolism, such as calcium, phosphate, 1,25(OH)_2_D, PTH, and FGF23, regulate each other via a feedback mechanism *in vivo*. FGF23 affects phosphate handling through its role in kidney tubules. Therefore, parathyroid tissue-specific ablation of αKlotho or FGFRs is required to resolve this issue and determine the unbiased function of FGF23 in the parathyroid glands. Using mice with parathyroid-specific knockout of *αKlotho* or *Fgfr* genes, we have shown here that FGF23 contributes to the induction of secondary hyperparathyroidism associated with CKD, and that the function of FGF23 in the parathyroid glands is mediated by the αKlotho/FGFR complex. FGF23 increases parathyroid cell proliferation and long-term PTH secretion. Some previous studies^11^,^12^ showed a suppressive effect of FGF23 on PTH secretion *in vivo* and *in vitro*, but these studies were designed to measure PTH secretion within 12 h *in vitro* or 24 h *in vivo*. Thus, the effect of FGF23 on parathyroid cell proliferation was not considered.

Our study focused on the long-term effect of FGF23 on the parathyroid glands *in vivo* and *in vitro*. With regard to the effect of extracellular calcium, classic *in vivo* studies showed that low calcium stimulates PTH synthesis and secretion as a result of its short- and long-term effects, and also enhances parathyroid cell proliferation, which is considered a long-term effect^1^,^5^. The effect of low calcium on PTH synthesis and secretion in the acute phase (i.e. within minutes or hours) has also been confirmed in *in vitro* studies that excluded the secondary effects of other factors affected by calcium concentration. Although low extracellular calcium has the same effect in both its acute phase and its chronic phase (increasing PTH secretion in both), FGF23 suppresses PTH secretion in the short term but increases secretion in the longer term. FGF23 signalling appears to be linked to the suppression of PTH secretion as an acute effect on parathyroid glands^11^,^12^, but is linked to cell proliferation through the activation of the ERK/MAPK pathway^31^. In our long-term study, CKD model mice showed highly proliferative parathyroid glands and high circulating levels of PTH. Furthermore, the specific ablation of *αKlotho* or *Fgfr* genes from the parathyroid glands reduced parathyroid cell proliferation and circulating PTH levels. These *in vivo* findings were supported by our results from long-term culture studies *in vitro*. Thus, we conclude that the long-term effect of FGF23 is to enhance the circulating levels of PTH by causing parathyroid cells to proliferate.

In our CKD mice, heminephrectomy plus 12 weeks of a high-phosphate diet resulted in impaired kidney function accompanied by high phosphate and low calcium plasma levels. A previous study used a kidney-disease model mouse which was induced by adenine ingestion^32^; however, this model appeared to represent acute kidney disease rather than CKD. The increase of serum phosphate was moderate in this model, and serum creatinine levels returned to normal after 3 to 4 weeks. Although the authors did not measure serum FGF23 levels, they showed an increase of serum PTH levels without any effect of *αKlotho* ablation in the parathyroid glands. We suspect that a longer period of CKD might be required to observe the secondary hyperparathyroidism associated with cell proliferation in the parathyroid glands. Nonetheless, FGFR and αKlotho expression was not markedly reduced in our CKD mice as compared with patients with longstanding CKD^20^. This might be explained by the extent of the decrease (45–65%) in creatinine clearance and the period (12 weeks) of kidney failure in our CKD mice.

PTH plays an important role in mineral metabolism. Under physiological conditions, PTH production and secretion as well as parathyroid cell proliferation are regulated by circulating calcium via the CaSR^1^. CaSR expression was slightly lower in our CKD mice, but it was not significantly different between the non-cKO and cKO groups, suggesting that CaSR expression is not the critical factor for explaining the difference in plasma PTH levels between these two groups.

The active form of vitamin D also suppresses the function of the parathyroid glands^4^. It is notable that plasma 1,25(OH)_2_D levels were elevated in our CKD model mice. Similar data were reported previously in nephrectomised rats^33^ and mice^34^. In addition to this, 5/6 nephrectomy resulted in hypercalcaemia, hyperphosphataemia, high serum PTH levels, and high FGF23 levels in mice fed a regular diet^34^. The remnant kidney retains sufficient vitamin D-activating capacity to compensate for, or even to over-compensate for, the loss of tissue expressing Cyp27b1. In our study, the high-phosphate and low-calcium diet might have caused hypocalcaemia and augmented the increase of plasma PTH levels, leading to higher 1,25(OH)_2_D levels. Furthermore, the decreased expression of *αKlotho* in the kidney probably lessened the effect of FGF23 signalling and reduced the suppressive effect of FGF23 on *Cyp27b1* expression. Our CKD mice showed higher kidney levels of both *Cyp27b1* and *Cyp24a1* expression as compared with non-CKD mice. Nevertheless, 1,25(OH)_2_D levels were not affected by cKO of *αKlotho* or *Fgfr* genes from the parathyroid glands, suggesting that 1,25(OH)_2_D was not the factor determining the plasma PTH levels in our CKD model mice. The expression of VDR in the parathyroid glands was unchanged and the plasma levels of 25(OH)D were not affected under the conditions we used in this study.

In the current study, the cKO-induced decrease of plasma PTH levels in early to mid-stage CKD mice had no effect on other parameters that are known to be modulated by PTH. The plasma levels of calcium, 1,25(OH)_2_D, and FGF23 in the CKD mice could have been reduced by any of our cKOs via decreased plasma PTH levels. However, decreases in these parameters are known to be compensated for to maintain homeostasis *in vivo*. A longer observation period could have shown deviation of some of these parameters. In other words, the current study was designed to see the effect of parathyroid-specific FGF23 signalling ablation on the parathyroid glands by minimising any other conditions that otherwise might have affected parathyroid function via feedback effects.

We showed here that the parathyroid glands express αKlotho and three FGFRs encoded by three genes: *Fgfr1, Fgfr2*, and *Fgfr3*^31^,^35^. In contrast to the small number of molecules composing receptor counterparts, FGF ligands consist of 22 members. Except for FGF11, −12, −13, and −14, FGF ligands are secreted proteins that mediate their biological functions by binding to FGFRs on the cell surface. Among the secreted FGF ligands, FGF15/19, FGF21, and FGF23 are regarded as endocrine factors. Their binding to FGFRs is limited by the expression of the FGFR co-receptors αKlotho and βKlotho; FGF23 requires αKlotho, whereas FGF15/19 and FGF21 require βKlotho for binding to the receptor and proper downstream signalling^36^. Since βKlotho is not expressed in the parathyroid glands and FGF ligands expressed in the glands are not specified, all the secreted FGF ligands, except FGF15/19 and FGF21, have the potential to activate FGF signalling in these cells. In the current study, we focused on examining the function of FGF23 by ablating FGFRs and αKlotho separately in the parathyroid glands. Single αKlotho knockout gave similar results to the simultaneous ablation of all four FGFRs. These findings corroborate the hypothesis that FGF23, among the secreted FGF ligands, is the major ligand affecting parathyroid function.

In conclusion, we have found that elevated FGF23 levels in CKD mice stimulate αKlotho/FGFR signalling in the parathyroid glands and induce cell proliferation in these glands, resulting in full-blown secondary hyperparathyroidism associated with CKD. These findings imply that activation of FGF23/αKlotho/FGFR signalling by any mechanism might be associated with cell proliferation in the parathyroid glands, which can be observed in cases such as those overexpressing αKlotho^17^.

## Methods

### Animal experiments

The protocols for animal experimentation were approved by the Institutional Animal Care and Use Committees of the RIKEN Kobe Branch and Wakayama Medical University, and all animal experiments were conducted in accordance with institutional guidelines.

### cKO of *Fgfr* and *αKlotho* genes from parathyroid cells

We generated three different cKO mouse lines: triple cKO mouse of *Fgfr1, Fgfr2*, and *Fgfr3 (Fgfr1–3*), quadruple cKO mouse of *Fgfr1, Fgfr2, Fgfr3*, and *Fgfr4 (Fgfr1–4*), and single cKO mouse of *αKlotho*. The *Fgfr1*^*flox*^ mouse^37^ was obtained from Dr Deng of NIDDK (Bethesda, MD); *Fgfr2*^*flox*^ mouse^38^ from Dr Ornitz of the Washington University Medical School (Missouri, USA); *Fgfr3*^*flox*^ mouse^39^ from Dr Chen of the Third Military Medical University (Chongqing, China). *αKlotho*^*flox*^ and *Fgfr4*^*flox*^ mice were generated in our laboratory using the protocols of RIKEN (Kobe, Japan), as described in Supplementary Information (including Supplementary Figs S1, S2 and S3 for *αKlotho*^*flox*^ mouse and Supplementary Figs S4, S5 and S6 for *Fgfr4*^*flox*^ mouse). These floxed mice were crossed with a transgenic *PTH-Cre* mouse^40^, which was purchased from the Jackson Laboratory (Bar Harbor, ME) to generate the cKO mice described above. Their genetic background was brought to that of C57BL/6 mice by back-crossing with C57BL/6 mice for at least ten generations. Non-cKO mice used for controls were littermates of each of the *Fgfr1–3, αKlotho*, and *Fgfr1–4* cKO mice.

### Generation of mice with secondary hyperparathyroidism

To generate the adult mouse model with long-term secondary hyperparathyroidism, we performed heminephrectomy at 8 weeks of age when the mice were on a regular diet for 4 weeks after weaning, followed by feeding for an additional 4 weeks with a regular diet and then 12 weeks of a high-phosphate diet^22^. To make controls for these mice, we generated mice fed with a regular diet after weaning without heminephrectomy. Our mouse diets were as follows: regular diet containing 1.07% calcium, 0.83% inorganic phosphate, and 137 IU/100 g vitamin D3; high-phosphate diet containing 0.4% calcium, 2.0% inorganic phosphate, and 137 IU/100 g vitamin D3.

### Tissue culture of parathyroid glands

Parathyroid glands from mice with a C57BL/6 genetic background were surgically dissected from thyro-parathyroid tissue under a stereomicroscope immediately after sacrifice, and a parathyroid gland with some attached thyroid tissue was coated with collagen gel (Collagen Gel Culturing Kit, #KP-7000; Nitta Gelatin Inc., Yao, Japan) and placed in a culture dish (#D11130H; Matsunami Glass Ind., Ltd., Kishiwada, Japan). The parathyroid gland was pre-incubated overnight with MEM containing 1 mM calcium, 10.14 mM phosphate, 2 mM L-glutamine, 10 mM HEPES (pH 7.4), ITS solution (#41400045; Thermo Fisher Scientific K.K., Yokohama, Japan), and penicillin-streptomycin (#15070063; Thermo Fisher Scientific K.K.), and the medium was replaced twice following a 2-h incubation in 200 μL medium for the measurement of baseline PTH secretion. The average PTH value in these two replaced media was taken to be representative of basal PTH secretion. Then, the tissue was incubated with 200 μL MEM containing 0.95 mM calcium with or without 100 ng/mL FGF23 (recombinant mouse FGF-23 protein; #2629-FG-025; R&D Systems, Inc., Minneapolis, MN) for 4 days with the medium exchanged every 24 h, and the medium on day 4 was collected for the measurement of PTH secretion. In some experiments, the effect of FGF23 was examined after a 1-h and 13-h incubation after measuring basal PTH secretion. The effect of a certain condition on PTH secretion was evaluated using the ratio of the amount of PTH secreted into the incubation medium in 1 h, 13 h, or 24 h (day 4) versus the amount of basal secretion in 2 h. The medium PTH concentration was determined using a mouse PTH (1–84) ELISA Kit (Immunotopics, Inc., Athens, OH). The cultured glands were treated for immunohistochemical studies immediately after removing the medium from the fourth-day culture.

### Immunohistochemistry

Paraffin-embedded paraformaldehyde-fixed thyro-parathyroid tissues were sectioned serially at 6-μm thickness. One in every six sections was stained with hematoxylin and eosin (HE) to determine the location of the parathyroid glands. Then, the sections containing the parathyroid glands were immunostained using the standard indirect immunofluorescence technique. The primary antibodies used in the study were for PTH (sc-9676; Santa Cruz Biotechnology, Dallas, TX), Ki67 (ab16667; Abcam, Cambridge, UK), phosphorylated ERK1/2 (#4370; Cell Signaling Technology, Danvers, MA), CaSR (MA1-934; Thermo Fisher Scientific K.K.), VDR (sc-1009; Santa Cruz Biotechnology), αKlotho (AF1819; R&D Systems), FGFR1 (sc-121; Santa Cruz Biotechnology), FGFR2 (sc-122; Santa Cruz Biotechnology), FGFR3 (sc-123; Santa Cruz Biotechnology), and FGFR4 (sc-9006; Santa Cruz Biotechnology) with DAPI (Thermo Fisher Scientific K.K.) co-staining for nuclei. The secondary antibodies used for indirect immunofluorescence staining were as follows: Alexa Fluor 568 or 488-conjugated anti-rabbit, -mouse, or -goat IgG produced in goat or donkey (Molecular Probes).

### Counting the number of Ki67-positive cells and apoptotic cells

Thyro-parathyroid glands taken from the left and right sides were sectioned serially at 6-μm thickness, and the location of the parathyroid glands was determined with HE staining. One in every six parathyroid sections was stained for Ki67 and DAPI to detect Ki67+ cells or for segmented DNA using the TdT-mediated dUTP nick end labelling method with background nuclear staining by hematoxylin to detect apoptotic cells (*In situ* Apoptosis Detection Kit, #MK500; Takara Bio Co. Ltd., Tokyo, Japan). Micrographs were taken with a BZ-9000 microscope (Keyence, Osaka, Japan) using a 40× objective lens. The total number of positively stained cells was counted using BZ-X analyser software that is incorporated into the BZ-9000 microscope system. The ratio of the number of Ki67+ versus DAPI+ cells or the number of apoptotic versus hematoxylin+ cells was calculated in each stained section. The average ratio in sections from both the left and right glands was regarded as the value representing a single animal. For the cultured parathyroid glands, the average ratio in sections from each gland was regarded as the value representing the condition.

### Biochemical analyses

Blood was drawn into a heparinised syringe, and plasma was separated by centrifugation at 4,000 rpm for 15 min. The plasma concentrations of calcium, phosphate, and creatinine were measured by an autoanalyser using an Aqua-auto Kainos Calcium Kit (Kainos Laboratories, Inc., Tokyo, Japan), a Determiner L IP II Kit (Kyowa Medex Co., Ltd., Tokyo, Japan), and an Aqua-auto Kainos Creatinine Kit (Kainos Laboratories, Inc.), respectively. The plasma levels of FGF23, 1,25(OH)_2_D, and PTH were quantified using an FGF23 ELISA Kit (Kainos Laboratories, Inc.), a 1,25(OH)_2_-Vitamin D-RIA-CT Kit (DIAsource ImmunoAssays, Louvain-la-Neuve, Belgium), and a Mouse PTH(1–84) ELISA Kit (Immunotopics, Inc), respectively. The plasma levels of 25(OH)D were measured using a 25OH-Vitamin D Total-RIA-CT Kit (DIAsource ImmunoAssays, Louvain-la-Neuve, Belgium). Urine was collected every 24 h for 3 consecutive days using individualised metabolic cages (#CM-10S; CLEA Japan, Inc., Tokyo, Japan), and measurement of the urine creatinine concentration was carried out by SRL, Inc. (Tokyo, Japan). Creatinine clearance was calculated as follows: urine creatinine concentration (mg/dL) × urine volume (mL/min)/plasma creatinine concentration (mg/dL). The average value based on 3 daily collections of urine was regarded as representing each mouse.

### Quantitative reverse transcription (RT) polymerase chain reaction (PCR)

Total RNA was extracted from tissues with TRI Reagent (Sigma-Aldrich), and the mRNA expression of *Opn, Vim, Ngal, Kim-1, αKlotho, Cyp27b1*, and *Cyp24a1* in the kidney was analysed using SYBR Green-included PCR following RT as described previously^41^. Relative mRNA expression levels were calculated by the delta-delta Ct method to normalise target gene mRNA to *Gapdh* as reported previously^41^.

The following primers were used: *Opn*, forward 5′-TCCAAAGAGAGCCAGGAGAG-3′ and reverse 5′-GGCTTTGGAACTTGCTTGAC-3′; *Vim*, forward 5′-CTGCACGATGAAGAGATCCA-3′ and reverse 5′-AGCCACGCTTTCATACTGCT-3′; *Ngal*, forward 5′-GAAATATGCACAGGTATCCTC-3′ and reverse 5′-GTAATTTTGAAGTATTGCTTGTTT-3′; *Kim-1*, forward 5′-CTGGAATGGCACTGTGACATCC-3′ and reverse 5′-GCAGATGCCAACATAGAAGCCC-3′; *αKlotho*, forward 5′-CCCGATGTATGTGACAGCCAATGG-3′ and reverse 5′-CTTGGGAGCTGAGCGATCACTAAG-3′; *Cyp27b1*, forward 5′-ATGGTGAAGAATGGCAGAGG-3′ and reverse 5′-TTAGTCGTCGCACAAGGTCA-3′; *Cyp24a1*, forward 5′-TGGTGCGGATTTCCTTTGT-3′ and reverse 5′-AGCTGTTTGCGGTCGTCTC-3′; and *Gapdh*, forward 5′-ACCCAGAAGACTGTGGATGG-3′ and reverse 5′-GGATGCAGGGATGATGTTCT-3′.

### Statistical analyses

Values are expressed as mean ± standard deviation (s.d.). Multiple values were compared by two-tailed analysis of variance followed by Tukey’s multiple comparison test. Comparisons between two values were performed by two-tailed Student’s t-test.

## Additional Information

**How to cite this article**: Kawakami, K. *et al*. Persistent fibroblast growth factor 23 signalling in the parathyroid glands for secondary hyperparathyroidism in mice with chronic kidney disease. *Sci. Rep.* **7**, 40534; doi: 10.1038/srep40534 (2017).

**Publisher's note:** Springer Nature remains neutral with regard to jurisdictional claims in published maps and institutional affiliations.

## Supplementary Material

Supplementary Information
