# Supplementary material for: Persistent fibroblast growth factor 23 signalling in the parathyroid glands for secondary hyperparathyroidism in mice with chronic kidney disease
Source: Sci Rep. 2017 Jan 17;7:40534. doi: 10.1038/srep40534 (PMC5240111; doi:10.1038/srep40534)
Supplement: Supplementary Information [file srep40534-s1.pdf]

Supplementary Information

Title:

Persistent fibroblast growth factor 23 signalling in the parathyroid glands for secondary hyperparathyroidism in mice with chronic kidney disease

Authors:

Kazuki Kawakami[*,1], Ai Takeshita[*,1], Kenryo Furushima[1], Masayasu Miyajima[2], Ikuji Hatamura[3], Makoto Kuro-o[4], Yasuhide Furuta[5] and Kazushige Sakaguchi[1]

Affiliation:

[1]Department of Molecular Cell Biology and Molecular Medicine, Institute of Advanced Medicine, Wakayama Medical University, 811-1 Kimiidera, Wakayama 641-8509, Japan

[2]Laboratory Animal Center, Wakayama Medical University, 811-1 Kimiidera, Wakayama 641-8509, Japan

[3]Division of Molecular Medicine, Kansai University of Health Sciences, Kumatori, Japan

[4]Center for Molecular Medicine, Jichi Medical University, Tochigi, Japan

[5]Animal Resource Development Unit and Genetic Engineering Team, RIKEN Center for Life Science Technologies, 2-2-3 Minatojima Minami-machi, Chuou-ku, Kobe 650-0047, Japan

Generation of *αKlotho* conditional knockout mice

The *αKlotho* conditional knockout (cKO) mice (Accession No. CDB1170K: http://www2.clst.riken.jp/arg/mutant%20mice%20list.html) were generated as described previously (http://www2.clst.riken.jp/arg/Methods.html). To construct a targeting vector, genomic fragments of the *αKlotho* locus were obtained from a mouse BAC clone (B6Ng01-086G19; RIKEN BRC, Tsukuba, Japan). Three genomic segments surrounding exon 2 of *αKlotho*—the 5′ arm, the floxed-out body, and the 3′ arm—were individually subcloned into a minimal vector using the BAC clone and a BAC subcloning kit (#K003; Gene Bridges GmbH, Heidelberg, Germany). A 1434 bp region containing exon 2 of the *αKlotho* gene was flanked by loxP sites (Supplementary Fig. S1a). The targeting vector was linearised by treatment with the restriction enzyme *Asc*I and introduced into HK3i, a C57BL/6-based ES cell line [1], by electroporation.

The PCR primers used with the BAC subcloning kit for subcloning the 5′ arm,

floxed-out body, and 3′ arm were as follows. A PCR kit (GoTaq® Green Master Mix, M7123; Promega) was used for this purpose. The 5′ arm was cloned using the forward primer 5′-ACATCCCAGGGGCCAGACTCAAGAGCTCACTTTGTCGTGACTATTCCTTCGTCGACGCTCTCCTGAGTAGGACAAATC-3′ and reverse primer 5′-ACTCCAACAACTGGATTCTATTTGGAAACTCCTAGTCTCTTAGGACAAAAGCTAGCTCACAGCTTGTCTGTAAGCGGATG-3′; the floxed-out body was cloned using the forward primer 5′-CCAATCTCCCATCTGTGGCTGTACGAGGAATTTCCAAATAGGATTATGTGCCGCGGGCTCTCCTGAGTAGGACAAATC-3′ and reverse primer 5′-CATTTGGGCCCTTCAGAAGCTTAATTTTCCAAGTACCTTAAAAGGAACTTGTTTAAACTCACAGCTTGTCTGTAAGCGGATG-3′; and the 3′ arm was cloned using the forward primer 5′-AATTGTTTTAAATATTAATCTTGTTTTATTGTCTTATCAGCAAATTATCAGCGGCCGCGCTCTCCTGAGTAGGACAAATC-3′ and reverse primer 5′-GTAACCAGACCCAAGTGAACCACACGGTTCTGCACTTCTACCGCTGCATGGCGCGCCTCACAGCTTGTCTGTAAGCGGATG-3′.

Targeted ES clones were selected by Sothern blotting and PCR using the

probes and primers described below, and microinjected into 8-cell stage ICR embryos. These injected embryos were transferred into pseudopregnant ICR females. The resulting chimaeras were bred with C57BL/6 mice, and heterozygous offspring were identified by Southern blotting (Supplementary Fig. S1b) and PCR (Supplementary Fig. S2) using the same probes and primers used for selecting targeted ES clones.

The probes used in the Southern blotting were as follows: the 5′ probe (1002 bases) was generated by PCR using the forward primer 5′-AGGTATCCTCCTTGAGACAGTGG-3′ and reverse primer 5′-GTTAGATAGGAAGTACCCATCGT-3′ with mouse genomic DNA as a template; the 3′ probe (1122 bases) was generated by PCR using the forward primer 5′-TCACCATGAACGAGCCAAACACA-3′ and reverse primer 5′-GGGAGGCCTATCAAGAGATAGAG-3′ with mouse genomic DNA as a template; and the Neo probe (877 bases) was generated by PCR using the forward primer 5′-CATTCTGCACGCTTCAAAAGCGCACG-3′ and reverse primer 5′-ACTCGTCAAGAAGGCGATAGAAGGCG-3′ with the targeting vector plasmid DT-ApA/conditional KO FW as a template.

The primers used for genotyping PCR (GoTaq® Green Master Mix, M7123; Promega) were the forward primer KL-F1 (5′-GGATGGTGGGCCAAGATACTAAGG-

3′) and the reverse primer KL-R1 (5′-CAATCTCCCATCTGTGGCTGTACG-3′) for the wild-type (WT) allele, and the forward primer KL-F1 and the reverse primer Neo-R (5′-AATTAAGGGCCAGCTCATTCCTCC-3′) for the targeted allele (Kfn allele), yielding 226 bp and 325 bp products, respectively. The PCR conditions were as follows: an initial cycle at 94 °C for 2 min, 35 cycles at 94 °C for 30 s, 60 °C for 30 s, and 72 °C for 1 min, and a final cycle at 72 °C for 10 min. The heterozygous mice were then crossed with C57BL/6 mice carrying the CAG-FLPe transgenic gene encoding flippase (RIKEN BRC, Tsukuba, Japan) to eliminate the neomycin-resistance gene flanked with two *Frt* sequences. The *αKlotho* flox allele (Kf allele) was identified by PCR using the forward primer KL-F1 and reverse primer KL-R1, yielding a 402 bp product.

To confirm whether the floxed allele can be precisely recognised by Cre recombinase, we mated the floxed *αKlotho* mice with a transgenic *CAG-Cre* mouse ubiquitously expressing Cre driven by the chicken β-actin promoter/enhancer to generate *αKlotho* KO mice ($\alpha Klotho^{flox/flox}$*;CAG-Cre*) [2]. *αKlotho* KO was identified by PCR using the forward primer KL-F1 and reverse primer KL-R2 (5′-CAATGTAGATGAGCGACTGCGAGTCC-3′), yielding a 519 bp product (Supplementary Fig. S2a and b). Furthermore, RNA was extracted from the kidney of WT, heterozygous, and KO mice, and the presence or absence of exon 2 of the *αKlotho*

gene was examined with RT-PCR using reverse transcriptase (High-Capacity Reverse Transcription Kits, #4368814; Applied Biosystems) and a PCR kit (GoTaq® Green Master Mix, M7123; Promega). The primers used were as follows: a forward primer in exon 1 (KL-E1, 5′-GGTCAGGTCAAGTACTGGATCACC-3′), a forward primer in exon 2 (KL-E2, 5′-AACCTGAGGCAGCTTCTGTCTTGG-3′), and a reverse primer in exon 3 (KL-E3, 5′-GACTTTGGCAACAGCTCCTTGTCC-3′) (Supplementary Fig. S3a and b). RT-PCR using the KL-E1 and KL-E3 primers produced a single 796 bp band in WT mice, double 796 and 252 bp bands in heterozygous mice, and a single 252 bp band in KO mice. These mice exhibited the same phenotypes (body size, plasma mineral concentrations, and kidney function) as those of the classic systemic *αKlotho* KO mice (Supplementary Fig. S3c and d) [3].

Generation of *Fgfr4* conditional knockout mice

The *Fgfr4* cKO mice (Accession No. CDB1190K: http://www2.clst.riken.jp/arg/mutant%20mice%20list.html) were generated as described previously (http://www2.clst.riken.jp/arg/Methods.html). To construct a targeting vector, genomic fragments of the *Fgfr4* locus were obtained from a mouse BAC clone (B6Ng01-346E16; RIKEN BRC, Tsukuba, Japan). Briefly, three genomic segments

surrounding exon 6 of *Fgfr4*, the 5′ arm, the floxed-out body, and the 3′ arm, were individually subcloned into a minimal vector using the BAC clone and a BAC subcloning kit (#K003; Gene Bridges GmbH, Heidelberg, Germany). These segments were assembled into the targeting vector plasmid DT-ApA/conditional KO FW (http://www2.clst.riken.jp/arg/cassette.html). Eventually, a 753 bp region containing exon 6 of the *Fgfr4* gene was flanked by loxP sites (Supplementary Figs. S4 and S5). The nucleotide sequence of the three segments in the final vector was confirmed by DNA sequencing. The targeting vector was linearised by treatment with the restriction enzyme *Asc*I and introduced into HK3i, a C57BL/6-based ES cell line [1], by electroporation.

The PCR primers used with the BAC subcloning kit for subcloning the 5′ arm, floxed-out body, and 3′ arm were as follows. The 5′ arm was cloned using the forward primer 5′-CCCTCCACCCAACCACTCCCGCCGCGCGGGCGCCAAGGTCCCCGGGACAGGTCGACGCTCTCCTGAGTAGGACAAATC-3′ and reverse primer 5′-TCTTCTTAACTCCTAAACAATGCCTCCAGCCCAACAGAGACACTTCTTACGCGGCCGCTCACAGCTTGTCTGTAAGCGGATG-3′; the floxed-out body was cloned using the forward primer 5′-

CCTGAGAGGATTAGGCCAAAGCCAGAGACTCCTGAACAGAGAAAGAGGCTGTTTAAACTGGATATCGCTCTCCTGAGTAGGACAAATC-3′ and reverse primer 5′-AAATAAACCTTTTATAAAAAAAAAGAATGTAGTGCATTTGTGGGCAGTATCCGCGGTCACAGCTTGTCTGTAAGCGGATG-3′; and the 3′ arm was cloned using the forward primer 5′-GAATGTCCACACCCTGTCCCCTAGCCCCACAAATGTTGCACCCATGGCATACTAGTCGATATTTAAATGCTCTCCTGAGTAGGACAAAT-3′ and reverse primer 5′-TCCCTAGCATTCTTCCCTTCCTCACTCCCTCCACTCTGCACAGCTGGAGCGTCGACTCACAGCTTGTCTGTAAGCGGATG-3′.

We screened the ES cells electroporated with the target vector for recombinant cells. More than ten recombinant ES clones were selected by performing Southern blotting with the 5′ and 3′ probes of the region that are described below, and PCR using several pairs of primers described below (primers FR4-F1, FR4-R1, FR4-F2, FR4-R2, and Neo-F) encompassing the targeted region.

The probes used in Southern blotting were as follows: the 5′ probe (794 bases) was generated by PCR using the forward primer 5′-ATAGCTAGGAGCTCTGCAGATGGC-3′ and reverse primer 5′-CCTTCTTCTGCTGTGTCTGAGAGC-3′ with mouse genomic DNA as a template; the

3′ probe (693 bases) was generated by PCR using the forward primer 5′-CTACATCCCTAGCCCGAAAGGCTTC-3′ and reverse primer 5′-ACAGATGGGGATCTGCAGGGTTAGC-3′ with mouse genomic DNA as a template; and the Neo probe (877 bases) was generated by PCR using the forward primer 5′-CATTCTGCACGCTTCAAAAGCGCACG-3′ and reverse primer 5′-ACTCGTCAAGAAGGCGATAGAAGGCG-3′ with the targeting vector plasmid DT-ApA/conditional KO FW as a template.

Targeted ES clones were microinjected into 8-cell stage ICR embryos, and the injected embryos were transferred into pseudo-pregnant ICR females. The resulting chimeric mice were bred with C57BL/6 mice, and heterozygous offspring were identified by Southern blotting and PCR (Supplementary Figs. S4 and S5). Southern blotting was carried out using the 5′ probe, 3′ probe, and Neo probe to confirm the presence of the correct inserted DNA fragment as shown in Supplementary Fig. S4. For PCR, the following primers were used: the forward primer FR4-F2 (5′-GGCTGAAGGAGCAGAAGTCTGC-3′) and reverse primer FR4-R2 (5′-GGAAGGACTTCTGAACACACAGC-3′) for the WT allele, and the forward primer Neo-F (5′-AATTAAGGGCCAGCTCATTCCTCC-3′) and reverse primer FR4-R2 for the targeted allele, yielding 454 bp and 256 bp products, respectively (Supplementary

Fig. S5a). The PCR conditions were as follows: an initial cycle at 94 °C for 2 min, 35 cycles at 94 °C for 30 s, 60 °C for 30 s, and 72 °C for 1 min, and a final cycle at 72 °C for 10 min. The heterozygous mice were then crossed with C57BL/6 mice carrying the CAG-FLPe transgenic gene encoding flippase (RIKEN BRC, Tsukuba, Japan) to eliminate the neomycin-resistance gene flanked with two Frt sequences. Subsequent several generations of mating these mice resulted in mice harbouring only the *Fgfr4* allele with the floxed exon 6. The floxed allele was identified by PCR using the forward primer FR4-F1 (5′-GAGTGTCAGATTCCCTAGGAACTGG-3′) and reverse primer FR4-R1 (5′-GACCCAGTAAAGCCAGGACAAGGAGAGG-3′) with a product of 467 bp, while the WT allele yielded a 266 bp product using the same pair of primers (Supplementary Fig. S5a and b).

To confirm whether the floxed allele can be recognised precisely by Cre recombinase, we mated the floxed *Fgfr4* mice with a transgenic *CAG-Cre* mouse ubiquitously expressing Cre driven by the chicken β-actin promoter/enhancer to generate *Fgfr4* KO mice ($Fgfr4^{flox/flox}$;*CAG-Cre*). Deletion of the floxed allele was detected by PCR using the forward primer FR4-F1 and reverse primer FR4-R2 with a product of 564 bp (cKO allele, Supplementary Fig. S5a and b). The PCR conditions used for primers FR4-F1, FR4-R1, and FR4-R2 were the same as those used for primers

FR4-F2, Neo-F, and FR4-R2.

Furthermore, RNA was extracted from the liver of WT, flox, and KO mice, and the presence or absence of exon 6 of the *Fgfr4* gene was examined with RT-PCR. The primers used were as follows: a forward primer in exon 5 (FR4-E5, 5′-CCTACCATCCACTGGCTCAAGG-3′), forward primer in exon 6 (FR4-E6, 5′-GGAGAACTCTCTGGGTAGCATTCG-3′), and reverse primer in exon 7 (FR4-E7, 5′-CGTGTTTCAGCCACTGTATGTGG-3′) (Supplementary Fig. S6a and b). RT-PCR using the forward primer FR4-E5 and reverse primer FR-E7 yielded a single 322 bp band in WT and flox mice, but a single 198 bp band in KO mice. The deletion of exon 6 was also confirmed by RT-PCR using the forward primer FR4-E6 and reverse primer FR-E7. In this RT-PCR, a single 182 bp band was detected in WT and flox mice, but not in the *Fgfr4* KO mice. The expression of FGFR4 protein in the liver was also confirmed to be knocked out in the *Fgfr4* KO mice. We then examined the mRNA expression levels of *Cyp7a1*, encoding a key enzyme for bile acid synthesis, in the liver by quantitative RT-PCR (iQ™ SYBR® Green Supermix, #1708880; BIO-RAD). FGFR4 regulates bile acid synthesis by suppressing *Cyp7a1* expression. *Fgfr4*$^{flox/flox}$;*CAG-Cre* mice showed a significant increase in liver *Cyp7a1* expression compared with WT mice as reported previously [4], whereas *Fgfr4*$^{flox/flox}$ mice expressed

Cyp7a1 at almost the same level as WT mice (Supplementary Fig. S6d).

Suppression of PTH secretion by calcium following long-term parathyroid culture

Parathyroid glands were cultured in dishes as collagen gel-coated tissues as described in the main text. The calcium concentration in the medium was changed to 0.95 mM or 1.5 mM after overnight preincubation in the same medium with 1.0 mM calcium and a subsequent 2-h incubation for the measurement of basal PTH secretion. The medium was exchanged every 24 h, and the secretion of PTH was quantified on the fourth day. The ratio of 24-h secretion versus 2-h basal secretion is shown in Supplementary Fig. S7 (5.8 ± 3.7 [mean ± SD], n = 4, at 0.95 mM calcium vs 2.5 ± 1.7, n = 5, at 1.5 mM calcium, $P < 0.05$). PTH secretion was suppressed by the high extracellular calcium concentration.

Viability of parathyroid cells after long-term culture

Parathyroid glands derived from non-cKO and cKO mice with either *Fgfr1-3* or *αKlotho* gene were cultured as described above, and incubated with or without FGF23 (100 ng/ml) for 4 days. The percentage of apoptotic cells is shown in Supplementary Fig. S8. The apoptotic cell ratio was less than 0.5% in all groups

examined, and did not significantly vary in the presence or absence of *Fgfr1–3* or *αKlotho* cKO, or after incubation with FGF23.

References for the supplementary information

1. Kiyonari, H., Kaneko, M., Abe, S. & Aizawa, S. Three inhibitors of FGF receptor, ERK, and GSK3 establishes germline-competent embryonic stem cells of C57BL/6N mouse strain with high efficiency and stability. *Genesis* **48**, 317-327, doi:10.1002/dvg.20614 (2010).

2. Araki, K., T. Imaizumi, K. Okuyama, Y. Oike and K. Yamamura (1997). "Efficiency of recombination by Cre transient expression in embryonic stem cells: comparison of various promoters." *J. Biochem.* **122**(5): 977-982.

3. Kuro-o, M. *et al.* Mutation of the mouse klotho gene leads to a syndrome resembling ageing. *Nature* **390**, 45-51, doi:10.1038/36285 (1997).

4. Ge, H., J. Zhang, Y. Gong, J. Gupte, J. Ye, J. Weiszmann, K. Samayoa, S. Coberly, J. Gardner, H. Wang, T. Corbin, D. Chui, H. Baribault and Y. Li (2014). Fibroblast growth factor receptor 4 (FGFR4) deficiency improves insulin resistance and glucose metabolism under diet-induced obesity conditions. *J. Biol. Chem.* **289**(44): 30470-30480.

Supplementary Figure S1

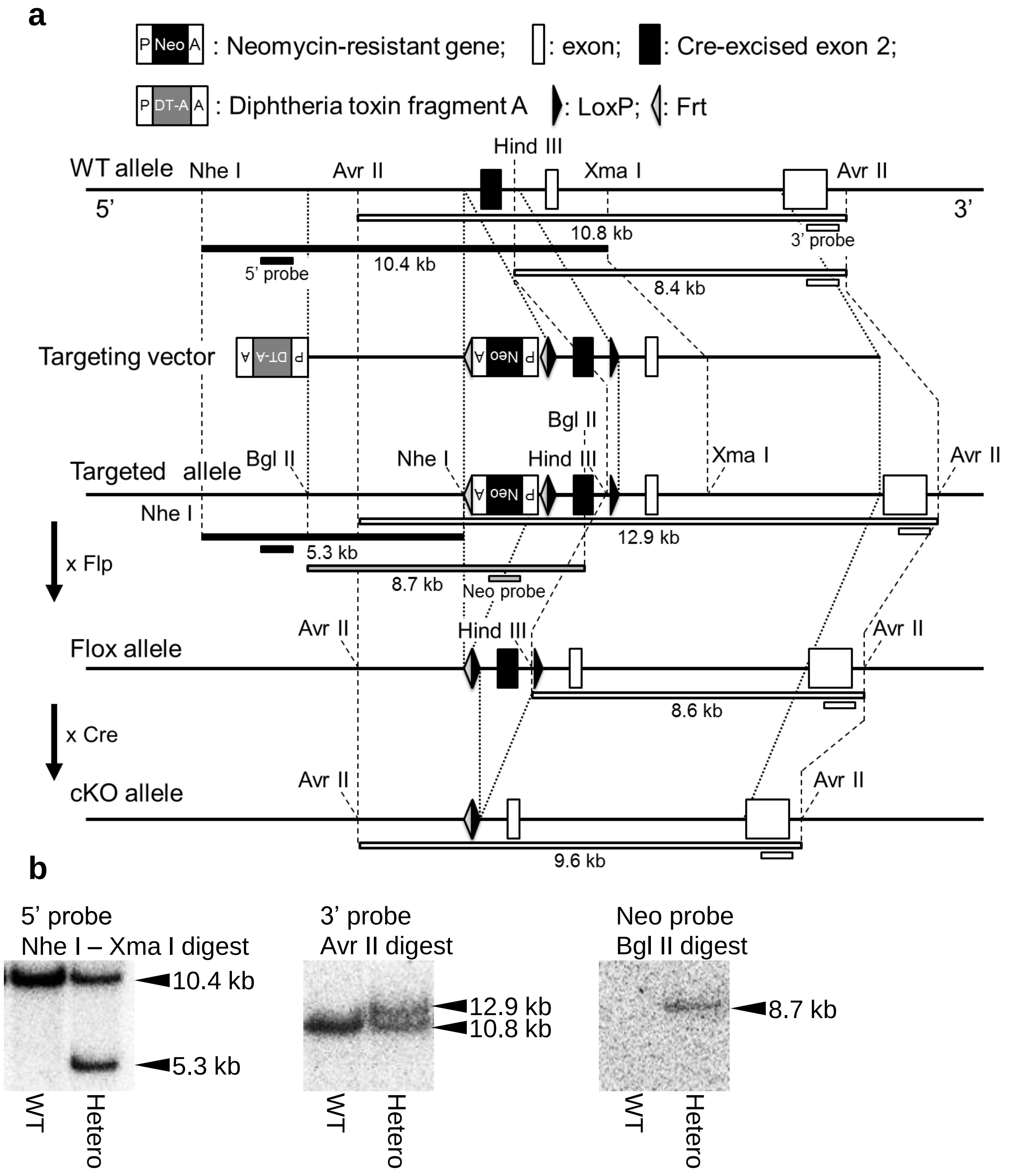

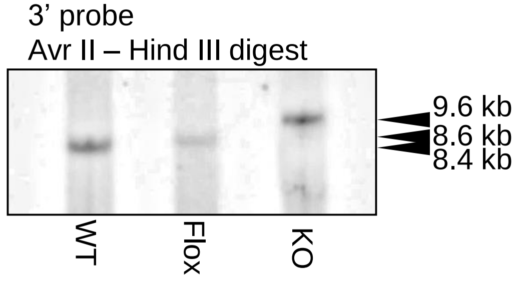

Supplementary Figure S1. Constructs for the conditional knockout of the *αKlotho* gene and Southern hybridization. (a) Schematic diagram of the *αKlotho* wild-type (*αKlotho^{WT}*), targeted (*αKlotho^{targeted}*), flox (*αKlotho^{flox}*), and conditional knockout (*αKlotho^{cKO}*) alleles for Southern hybridization. (b) Southern blot analysis of genomic DNA from WT (*αKlotho$^{WT/WT}$*), heterozygous (*αKlotho$^{flox/WT}$*), flox (*αKlotho$^{flox/flox}$*), and KO (*αKlotho$^{flox/flox}$;CAG-Cre*) mice. Genomic DNA was digested with *Nhe*I–*Xma*I, *Avr*II, and *Bgl*II, and hybridised with the 5' probe, 3' probe, and Neo probe, respectively. Genomic DNA from WT, flox, and KO mice was also digested with *Avr*II–*Hind*III, and hybridised with the 3' probe.

Supplementary Figure S2

a

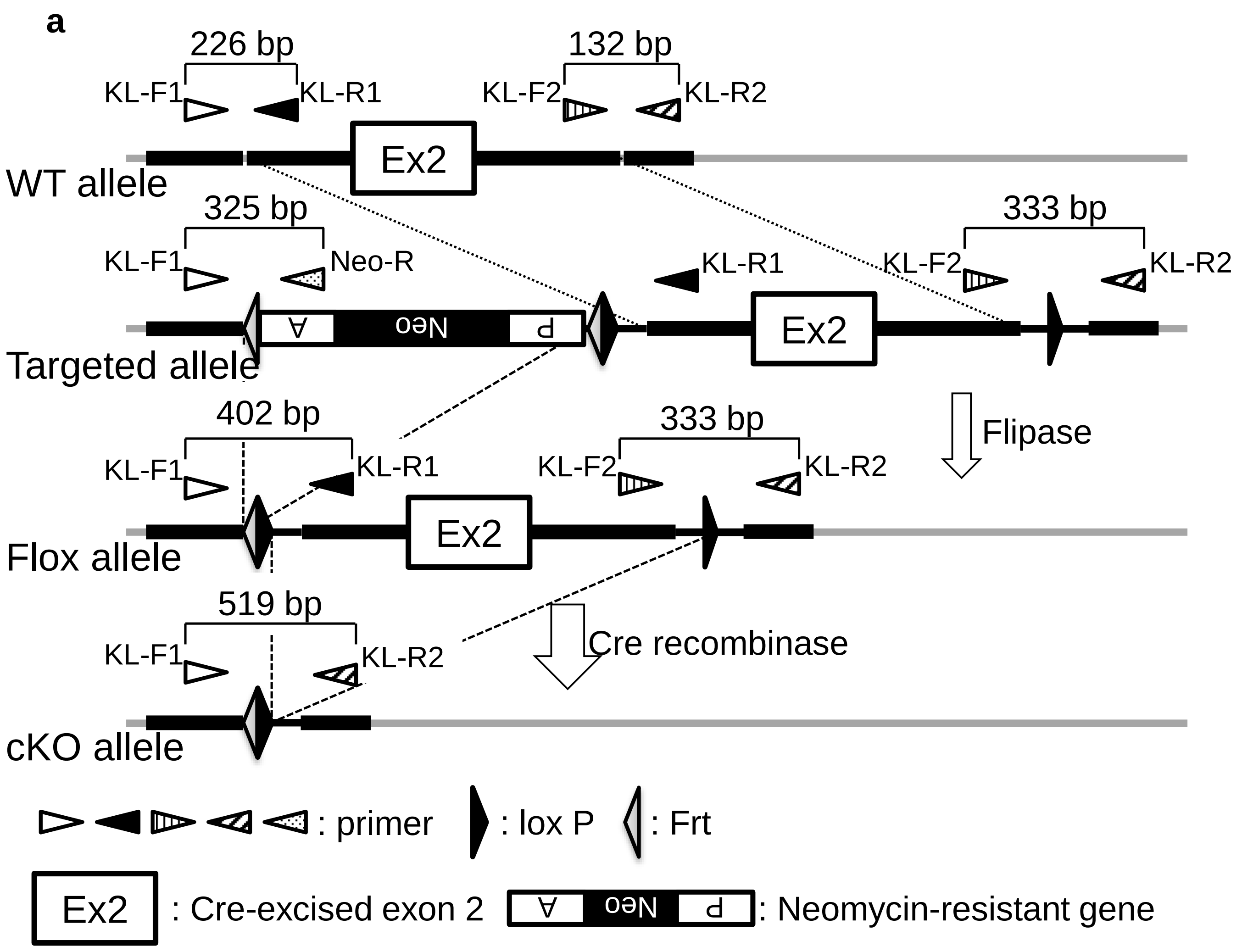

b

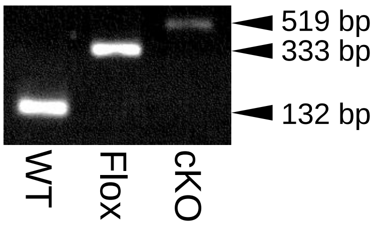

Supplementary Figure S2. Genotyping of *αKlotho* alleles by PCR.

(a) Schematic diagram *αKlotho*^{WT}, *αKlotho*^{targeted}, *αKlotho*^{flox}, and *αKlotho*^{cKO} alleles around exon 2.

(b) PCR genotyping of WT, flox, and cKO alleles with the forward primer KL-F1 or KL-F2 and the reverse primer KL-R2, producing 132, 333, and 519 bp bands, respectively.

Supplementary Figure S3

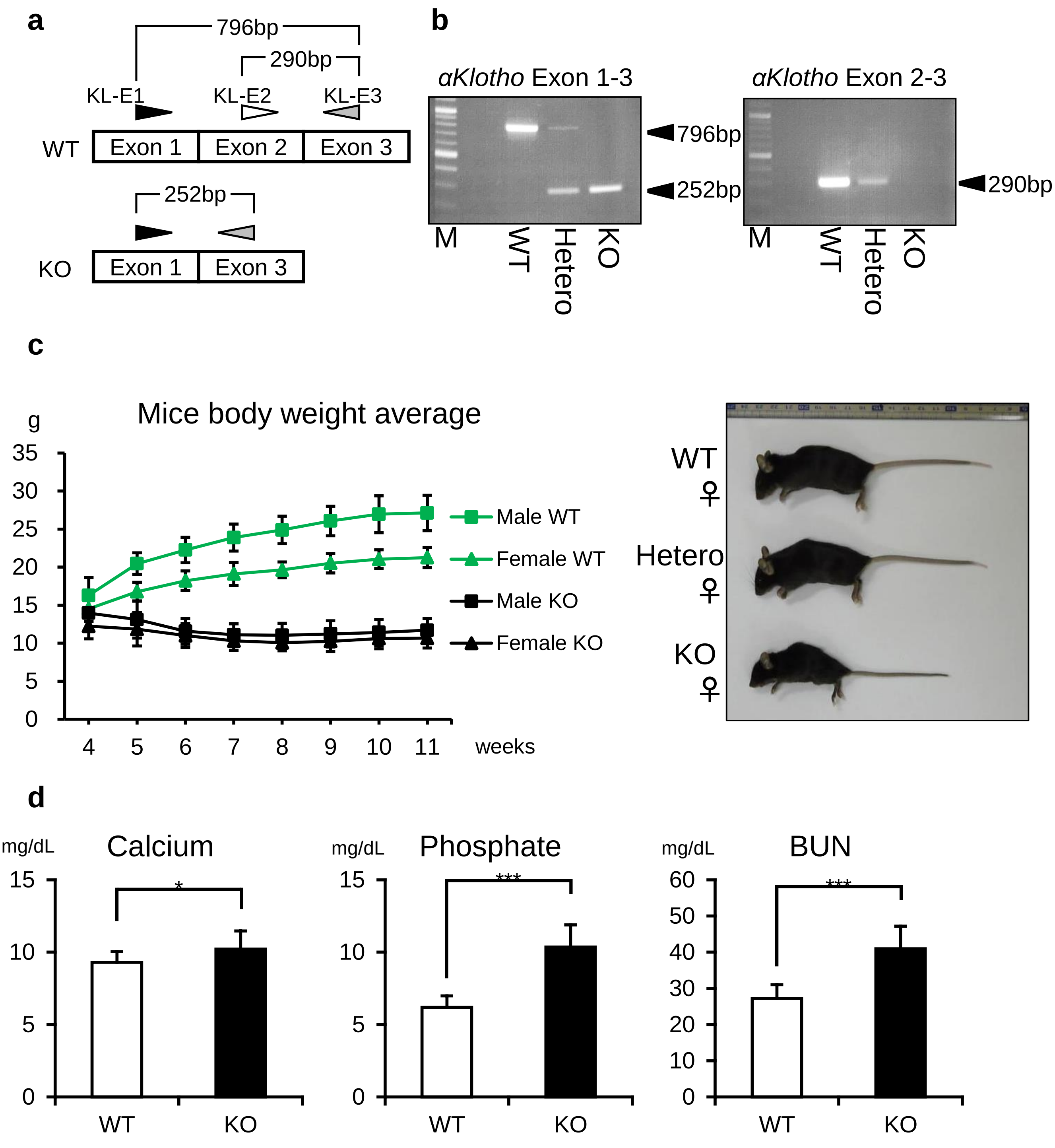

Supplementary Figure S3. Phenotypes of *αKlotho* gene-targeted mice.
(a) Schematic representation of *αKlotho* exons 1–3. In cKO mice, exon 2 was excised using Cre recombinase.
(b) RT-PCR products of kidney RNA extracted from WT, heterozygous, and KO mice. The forward primer KL-E1 or KL-E2 and the reverse primer, KL-E3 were used for detecting the inclusion or deletion of exon 2 in *αKlotho* mRNA. M: 100-bp ladder DNA size marker.
(c) Weight and gross appearance of WT, heterozygous, and KO mice. Male WT, n = 10; female WT, n = 18; male KO, n = 12; female KO, n = 13.
(d) Serum levels calcium, phosphate, and blood urea nitrogen (BUN) of WT and KO mice. WT, n = 13; KO, n = 12. *$p < 0.05$, ***$p < 0.001$.

Supplementary Figure S4

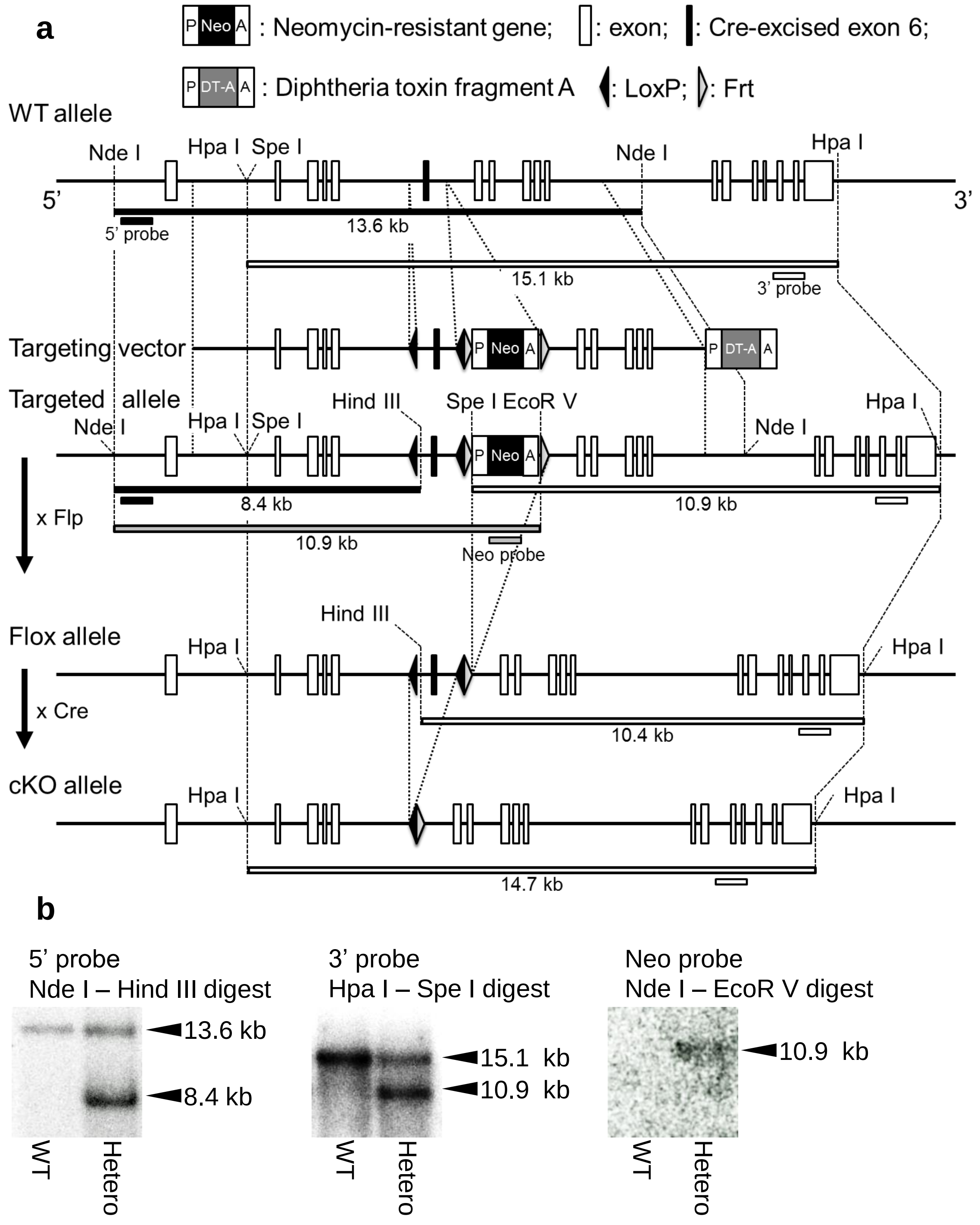

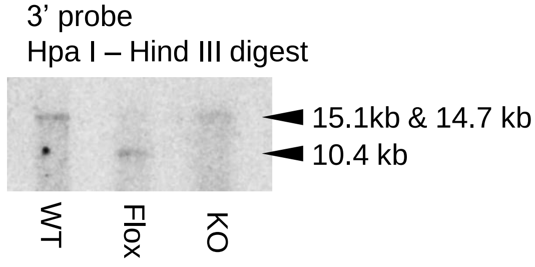

Supplementary Figure S4. Constructs for the cKO of the *Fgfr4* gene and Southern hybridization.

(a) Schematic diagram of the *Fgfr4* WT (*Fgfr4*^{WT}), targeted (*Fgfr4*^{targeted}), flox (*Fgfr4*^{flox}), and cKO (*Fgfr4*^{cKO}) alleles for Southern hybridization.

(b) Southern blot analysis of genomic DNA from WT (*Fgfr4*$^{WT/WT}$), heterozygous (*Fgfr4*$^{flox/WT}$), flox (*Fgfr4*$^{flox/flox}$), and KO (*Fgfr4*$^{flox/flox}$*;CAG-Cre*) mice. Genomic DNA was digested with *Nde*I–*Hind*III, *Hpa*I–*Spe*I, and *Nde*I–*Eco*RV, and hybridised with the 5′ probe, 3′ probe, and Neo probe, respectively. Genomic DNA from WT, flox, and KO mice was also digested with *Hpa*I–*Hind*III, and hybridised with the 3′ probe.

Supplementary Figure S5

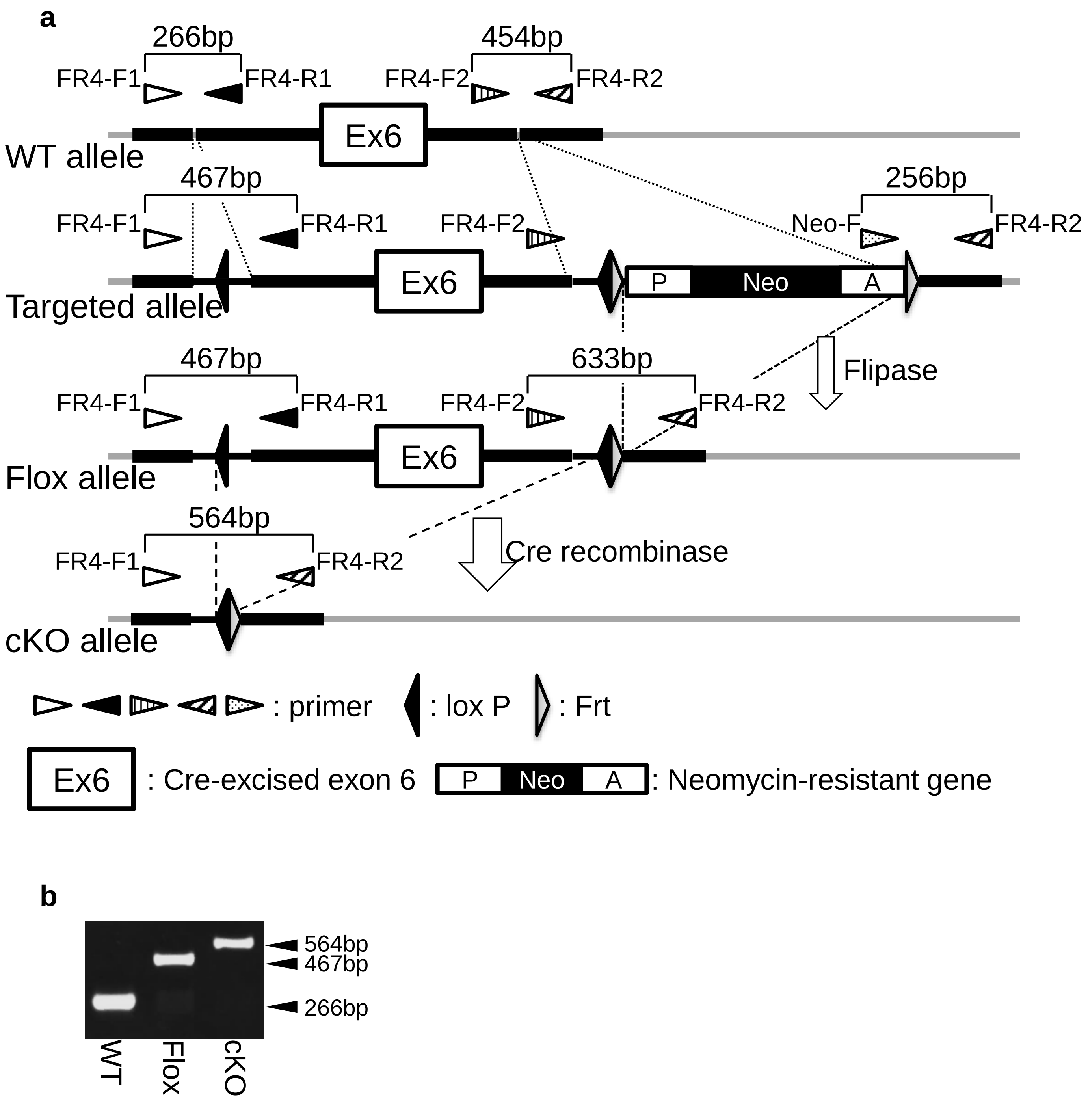

Supplementary Figure S5. Genotyping of *Fgfr4* alleles by PCR.
(a) Schematic diagram of the $\mathit{Fgfr4}^{WT}$, $\mathit{Fgfr4}^{targeted}$, $\mathit{Fgfr4}^{flox}$, and $\mathit{Fgfr4}^{cKO}$ alleles around exon 6.
(b) PCR genotyping of the WT, flox, and KO alleles with the forward primer FR4-F1 and reverse primer FR4-R1 or FR4-R2, producing 266, 467, and 564 bp bands, respectively.

Supplementary Figure S6

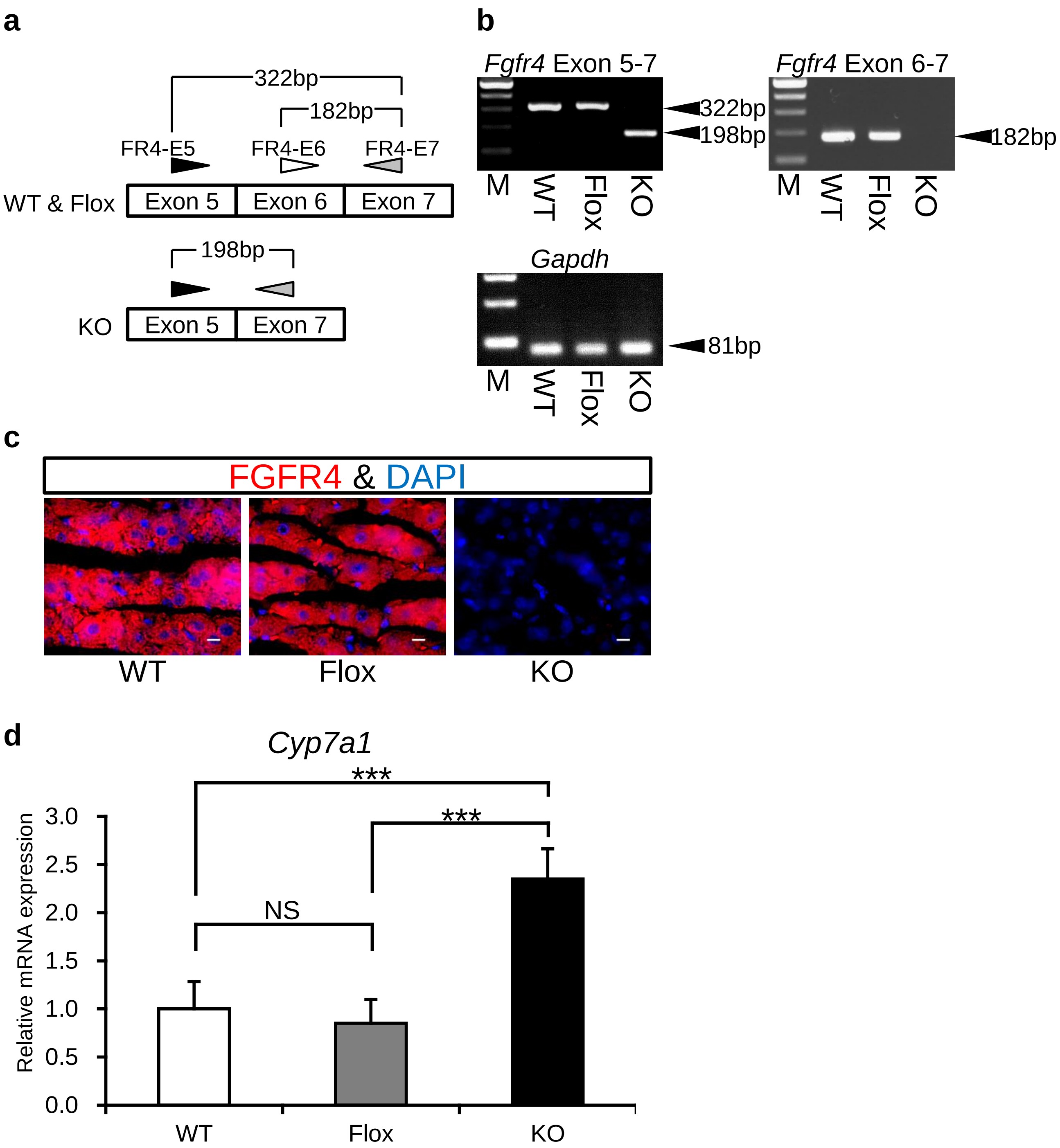

Supplementary Figure S6. Phenotypes of *Fgfr4* gene cKO mice.
(a) Schematic representation of *Fgfr4* exons 5–7. In cKO mice, exon 6 was excised using Cre recombinase.
(b) RT-PCR products of liver RNA extracted from WT, flox, and KO mice. The forward primer FR4-E5 or FR4-E6 and the reverse primer E7 were used to detect the inclusion or deletion of exon 6 in *Fgfr4* mRNA. *Gapdh* primers were used as a positive controls. M: 100-bp ladder DNA size marker.
(c) Immunofluorescence of FGFR4 in the liver from WT, flox, and KO mice. Red: FGFR4; blue: DAPI nuclear staining. Scale bars: 10 µm.
(d) Quantitative RT-PCR of *Cyp7a1* in liver from WT, flox, and KO mice. WT, n = 6; flox, n = 6; KO, n = 5. ***p < 0.001.

Supplementary Figure S7

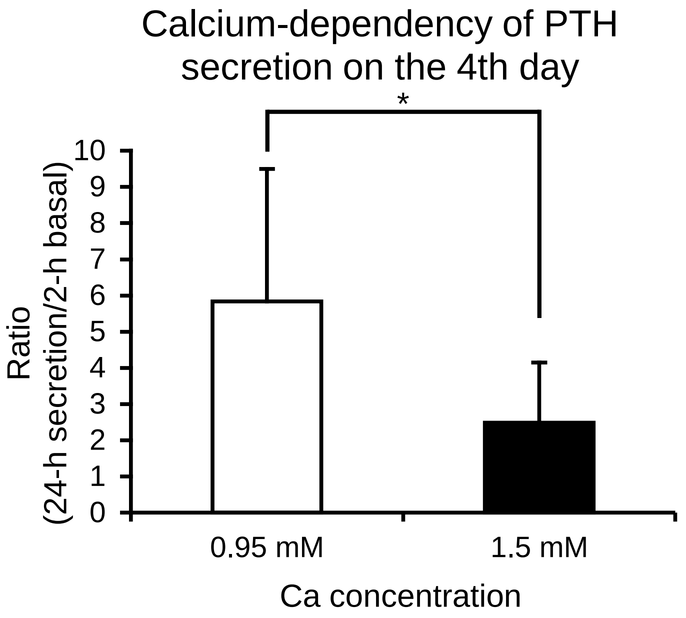

Supplementary Figure S7. Calcium-dependent suppression of PTH secretion from parathyroid glands cultured for 4 days. Parathyroid glands were cultured as described in the main text, except that the calcium in the medium was changed to two different concentrations (0.95 and 1.5 mM) following preincubation at 1.0 mM calcium. The ratio of 24-h PTH secretion on the 4th day of culture versus basal 2-h PTH secretion in 1.0 mM calcium is shown. The ratio representing incubation at 0.95 mM calcium was 5.8 ± 3.7 (mean ± SD), n = 4, and the ratio representing incubation at 1.5 mM calcium was 2.5 ± 1.7, n = 5. *P < 0.05.

Supplementary Figure S8

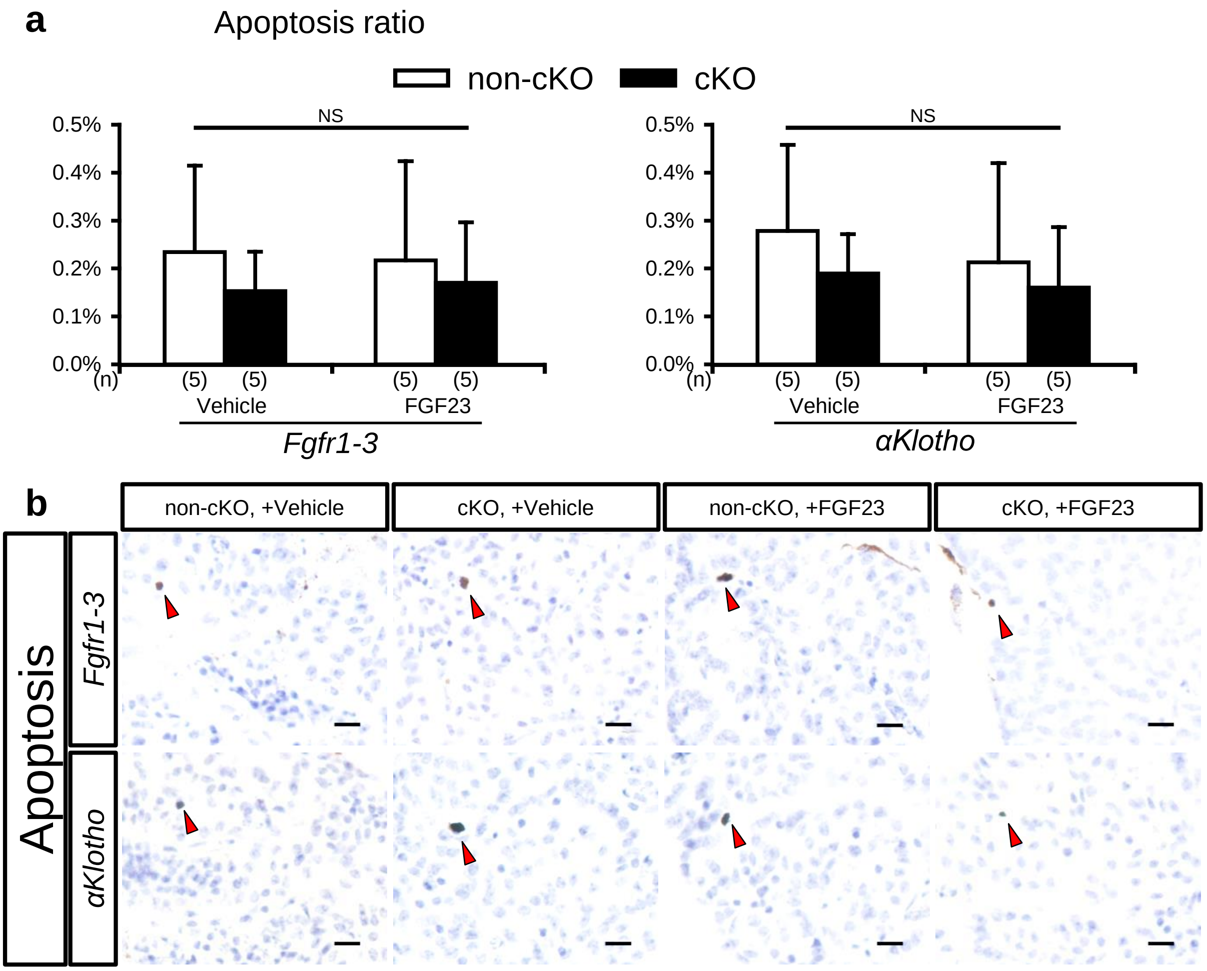

Supplementary Figure S8. Survival of parathyroid cells after long-term culture. (a) Percentage of apoptotic parathyroid cells at the end of long-term culture. Parathyroid glands were derived from the mice with non-cKO and cKO of the *Fgfr1-3* or *αKlotho* gene, and incubated with or without FGF23 (100 ng/ml) for 4 days following overnight preincubation. The number of apoptotic cells and total cell number were detected and counted as described in the Methods section of the main text. (b) Representative micrographs of staining for apoptotic cells in parathyroid tissue sections. Arrowheads indicate apoptotic cells. Scale bars: 10 µm. NS, not significantly different (P>0.05).
